# Gut microbiota of Pacific white shrimp (*Litopenaeus vannamei*) exhibits distinct responses to pathogenic and non-pathogenic *Vibrio parahaemolyticus*

**DOI:** 10.1128/spectrum.01180-23

**Published:** 2023-09-26

**Authors:** Yi-Ting Chang, Hao-Ting Ko, Ping-Lun Wu, Ramya Kumar, Han-Ching Wang, Hsiao-Pei Lu

**Affiliations:** 1 Department of Biotechnology and Bioindustry Sciences, College of Biosciences and Biotechnology, National Cheng Kung University, Tainan, Taiwan; 2 International Center for Scientific Development of Shrimp Aquaculture, National Cheng Kung University, Tainan, Taiwan; Hong Kong University of Science and Technology, Hong Kong

**Keywords:** acute hepatopancreatic necrosis disease (AHPND), gut microbiota, *Litopenaeus vannamei*, pathogen invasion, *Vibrio parahaemolyticus*

## Abstract

Acute hepatopancreatic necrosis disease (AHPND), a high-mortality-rate shrimp disease, is caused by specific *Vibrio parahaemolyticus* (Vp) strains with a plasmid encoding the PirAB^Vp^ toxins. As a bacterial pathogen, the invasion of AHPND-causing Vp might impose pressure on commensal microbiota in the shrimp gut, while the relationship between the pathogenesis of AHPND and the dysbiosis of gut bacterial communities remains unclear. Here we explored the temporal changes of shrimp gut microbiota in response to AHPND-causing and non-AHPND-causing Vp strains, with the non-infected controls as a baseline of the shrimp gut microbiota. The diversity and composition of bacterial communities from 168 gut samples (covering three treatments at seven time points with eight individuals per set) were investigated using 16S rRNA gene metabarcoding with high-throughput sequencing. The results showed that (i) species diversity of gut bacterial communities declined in Vp-infected shrimp, independent of the strain pathogenicity; (ii) taxonomic compositions of gut bacterial communities were significantly different between shrimp infected by AHPND-causing and non-AHPND-causing Vp strains; (iii) short-term (within 6 hours) compositional shifts in the gut microbiota were found only in AHPND-causing Vp-infected shrimp; (iv) the gut microbiota of AHPND-causing Vp-infected shrimp was enriched with genera *Photobacterium* and *Vibrio*, with a decline in *Candidatus* Bacilliplasma; and (v) functional predictions suggested the loss of normal metabolism due to compositional shifts in the gut microbiota. Our work reveals distinct features of community dynamics in shrimp gut microbiota, associated with pathogenic versus non-pathogenic *Vibrio* infections, providing a new perspective of the pathogenesis of AHPND.

**IMPORTANCE:**

Shrimp production is continually threatened by newly emerging diseases, such as AHPND, which is caused by specific Vp strains. Previous studies on the pathogenesis of AHPND have mainly focused on the histopathology and immune responses of the host. However, more attention needs to be paid to the gut microbiota, which acts as the first barrier to pathogen colonization. In this study, we revealed that shrimp gut microbiota responded differently to pathogenic and non-pathogenic Vp strains, with bacterial genera *Photobacterium* and *Vibrio* enriched in pathogenic Vp-infected shrimp, and *Candidatus* Bacilliplasma enriched in non-pathogenic Vp-infected shrimp. Moreover, functional predictions suggested that changes in taxonomic compositions would further affect normal metabolic functions, emphasizing the importance of sustaining an equilibrium in the gut microbiota. Several biomarkers associated with specific microbial taxa and functional pathways were identified in our data sets, which help predict the incidence of disease outcomes.

## INTRODUCTION

Pacific white shrimp (*Litopenaeus vannamei*) is one of the most widely farmed crustacean species with a high economic value in aquaculture (1), while its production is threatened by disease outbreaks in recent years (2). Among the diseases listed by the World Organization for Animal Health (founded as the Office International des Epizooties, OIE), acute hepatopancreatic necrosis disease (AHPND) is one of major diseases that severely threaten shrimp farming in Asia and Latin America (2).

Primary pathogenic agents of AHPND are unique strains of *Vibrio parahaemolyticus* (Vp), a Gram-negative bacterium commonly found in aquatic habitats (3). The AHPND-causing Vp strains contain an extrachromosomal plasmid encoding specific genes for PirA^Vp^ and PirB^Vp^ toxins, while this plasmid is not found in non-AHPND-causing Vp strains (4). The AHPND-causing Vp strains initially colonize the shrimp stomach and release the binary toxins from the stomach into the hepatopancreas, inducing sloughing of tubule epithelial cells followed by shrimp mortality (5 – 7). Since the histological hallmark of AHPND is hepatopancreatic pathognomonic lesions in the absence of any causative pathogen, it has been suggested that the pathology of the AHPND-causing strain is due to secreted toxins rather than the presence of the bacteria itself (6). However, during the terminal phase of AHPND, the hepatopancreatic tubules are surrounded by hemocytic capsules as a response to secondary bacterial infections, possibly caused by a vibriosis (8). Moreover, the presence of PirAB^Vp^ toxins could modulate the virulence of non-AHPND-causing *Vibrio* species and aggravate vibriosis (9). Although many efforts have been made, the pathogenesis of AHPND remains unclear and needs further investigation.

Aquatic crustaceans literally live in water with diverse microorganisms, making them susceptible to potential pathogens, including viruses, bacteria, fungi, and protists (10). In recent years, emerging evidence suggests that the gut microbiota plays a critical role in host physiological processes such as nutrient acquisition, immune activation, and initial defense against infection (11, 12). Gut microbiota comprises diverse commensal bacteria that provide intrinsic protection against pathogen colonization and stimulate the host immune responses, particularly during pathogen invasion (13). Once the balance of the gut microbiota is disrupted, the host may become more susceptible to the invading pathogens. In the aquaculture system, gut microbiota dysbiosis (the altered composition of bacterial communities) could significantly disrupt the stability of normal gut functions, leading to disease aggravation (14). Therefore, an understanding of the dynamics of the shrimp gut microbiota during the invasion of AHPND-causing Vp is crucial for a better understanding of the pathogenesis of AHPND.

Previous studies have mainly focused on the histopathology and immune responses of the shrimp host during AHPND infection (15). However, more attention needs to be paid to the gut microbiota, which acts as the first barrier to pathogen colonization. Some authors have shown that AHPND infection indeed has effects on shrimp gut microbiota (16 – 18). Significant differences in gut bacterial communities were detected between healthy and AHPND-infected shrimp, with an increase in some disease-specific bacterial taxa (16). Abundances of specific bacterial taxa have been reported to show a positive correlation with immune gene expressions of the host (19, 20), suggesting that the compositional changes of gut microbiota are vital in disease pathogenesis (21, 22).

For the invasion of an external microbe, competition for space and nutrients is crucial for successful colonization of gut ecosystems that already contain resident bacteria. Pathogenic bacteria have antagonistic interactions with the gut microbiota through the release of virulence factors (23, 24), while non-pathogens or probiotics might have neutral or commensal interactions with the gut microbiota (25, 26). The differences between AHPND-causing and non-AHPND-causing Vp in carbon source utilizations have been reported, implicating that they adopt distinct strategies to acquire resources in the shrimp gut (27). However, it remains unclear whether the gut microbiota responds differently to AHPND-causing and non-AHPND-causing Vp strains. Therefore, in this study, we aimed to characterize the dynamic responses of the gut microbiota during the invasion by both AHPND-causing and non-AHPND-causing Vp strains, as a reference for the pathogenesis of AHPND. We hypothesized that the colonization of both AHPND-causing and non-AHPND-causing Vp strains would result in changes in the shrimp gut microbiota, while these changes might differ in terms of diversity and composition.

Herein, we investigated the temporal changes of the gut microbiota during the infection with AHPND-causing and non-AHPND-causing Vp strains, with non-infected controls as a baseline of the shrimp gut microbiota. We collected a total of 168 shrimp gut samples, focusing on the early infection stage (0, 3, 6, 12, 24, 48, and 72 hours post immersion) to closely observe the responses of gut bacterial communities to the colonization of a bacterial pathogen in the shrimp stomach. We employed high-throughput sequencing of the 16S ribosomal RNA gene to characterize the dynamics of shrimp gut microbiota. We compared the diversity and composition of gut bacterial communities in shrimp infected with either AHPND-causing or non-AHPND-causing Vp strains to explore differential community responses mediated by bacterial interactions. Exploring dynamic community patterns of the gut microbiota during the course of the disease would provide further insights into the pathogenesis of AHPND.

## RESULTS

### Time-varying pattern of AHPND virulence in shrimp

**FIG 1:**
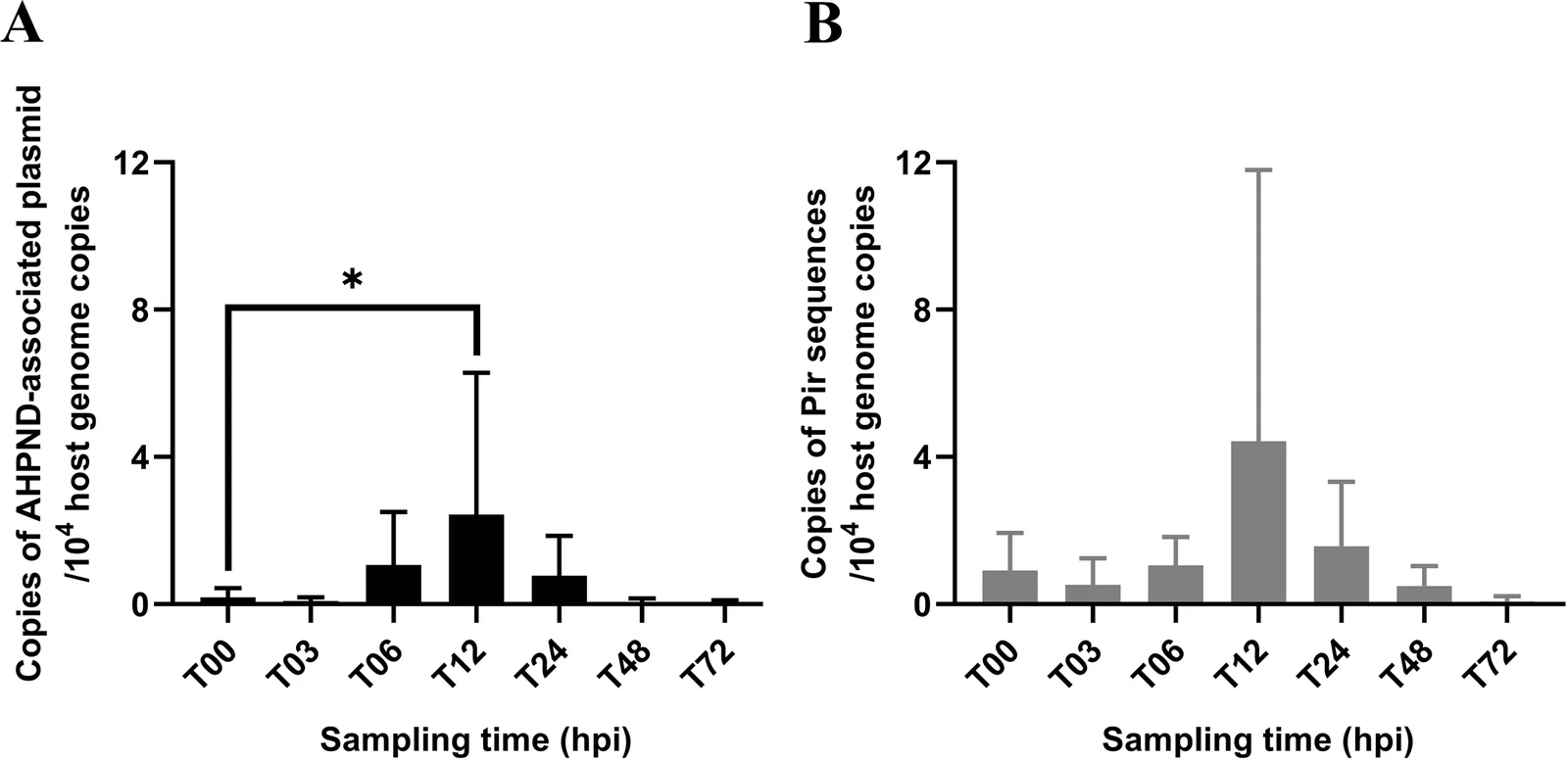
AHPND detection in 5HP-infected shrimp. The relative copies of AHPND-associated plasmid (**A**) and toxin gene (**B**) of pathogenic Vp, detected in shrimp individuals of the 5HP-infected group collected at different time points. The relative copies of AHPND plasmid and toxin gene were normalized against the shrimp genome copies. The sample size at each time point was *n* = 8, except for T72 (*n* = 5). Shrimp collected at T12 contained significantly higher numbers of AHPND-associated plasmid copies than those collected at T00. Statistical significance was calculated based on one-way analysis of variance with Dunnett’s test. *: p < 0.05.

The real-time PCR results showed that for shrimp in the 5HP-infected group, the copy numbers of the AHPND plasmid and toxin gene were highest at T12 (Fig. 1). The absence of AHPND-associated gene fragments was confirmed by PCR amplification for the S02-infected and tryptic soy broth (TSB)-treated groups. The mortality rate of shrimp in the 5HP-infected group was higher than that of shrimp in the S02-infected and TSB-treated groups (Fig. S1). Specifically, approximately 11% of mortality was observed between T06 and T24 in the 5HP-infected group, whereas very few shrimp individuals died in the S02-infected and TSB-treated groups. The majority of deaths in the 5HP-infected group occurred at T12, which was consistent with the presence of elevated levels of AHPND-associated genes at T12 (Fig. 1).

### Differences in α-diversity of gut microbiota

**FIG 2:**
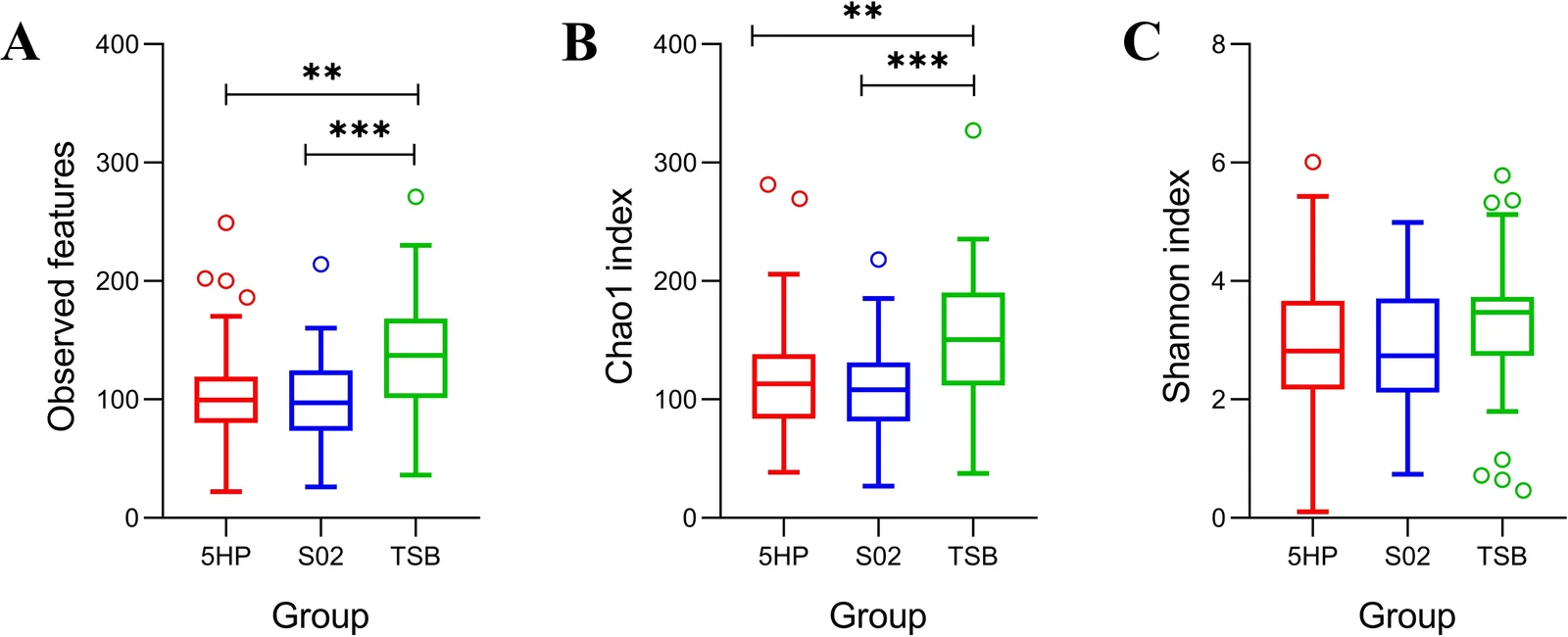
Differences in species diversity of gut microbiota among three experimental groups. Gut microbiota α-diversity was determined using three diversity indices, including (**A**) observed features, (**B**) Chao1, and (**C**) Shannon. Different colors in boxplots indicate gut microbiota samples from three distinct experimental groups: red indicates 5HP-infected (*n* = 56); blue indicates S02-infected (*n* = 54); and green indicates TSB-treated (*n* = 55) groups. The samples of the three experimental groups included all samples from all time points. The TSB-treated group showed significantly higher α-diversity in terms of observed features and Chao1 but not Shannon. Statistical significance was calculated based on the Kruskal-Wallis test and post hoc Dunn tests. **P < 0.01, ***P < 0.001.

**FIG 3:**
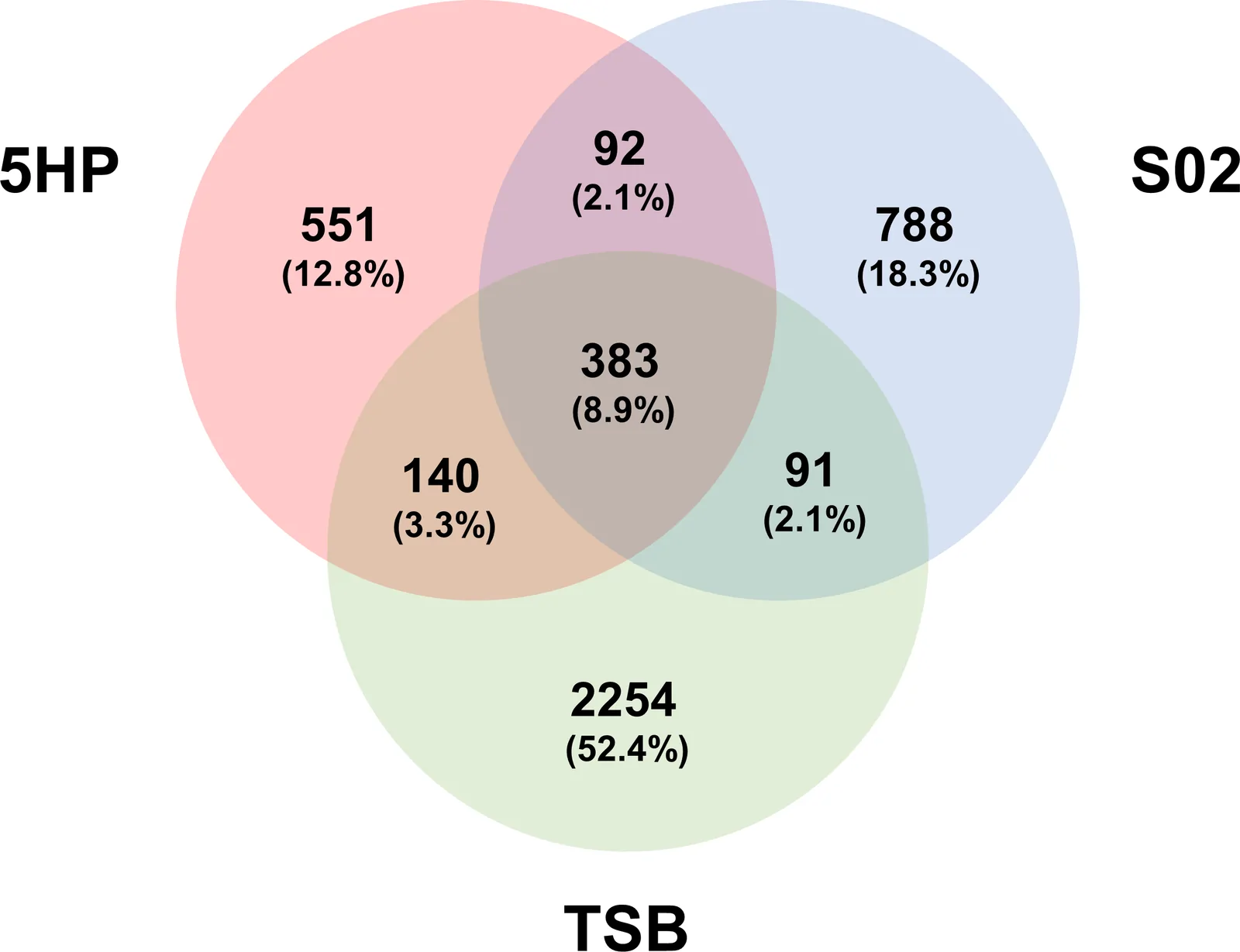
Amplicon sequence variant (ASV) Venn diagram of gut microbiota of three groups. Venn diagram showed the overlapping patterns of ASVs among three experimental groups from all time points: the red circle indicates 5HP-infected (*n* = 56); the blue circle indicates S02-infected (*n* = 54); and the green circle indicates TSB-treated (*n* = 55) groups.

Regarding species diversity of the shrimp gut microbiota, the bacterial communities of the 5HP-infected and S02-infected groups showed lower α-diversity values compared to the TSB-treated group, with significant differences in observed features and Chao1 indices but not in Shannon (Fig. 2). However, considering the temporal variation, the α-diversity values in the 5HP-infected and S02-infected groups showed no significant changes at different time points (Fig. S2). Focusing on the differences in richness among the three groups, the Venn diagram showed that 383 amplicon sequence variants (ASVs) were common to all groups, while each group contained its own unique ASVs, with 551 unique ASVs in the 5HP-infected group, 788 in the S02-infected group, and 2,254 in the TSB-treated group (Fig. 3). These results indicated that the invasion of both Vp 5HP and S02 strains would reduce the number of bacterial species (i.e., richness) in the shrimp gut microbiota.

### Differences in β-diversity of gut microbiota

**FIG 4:**
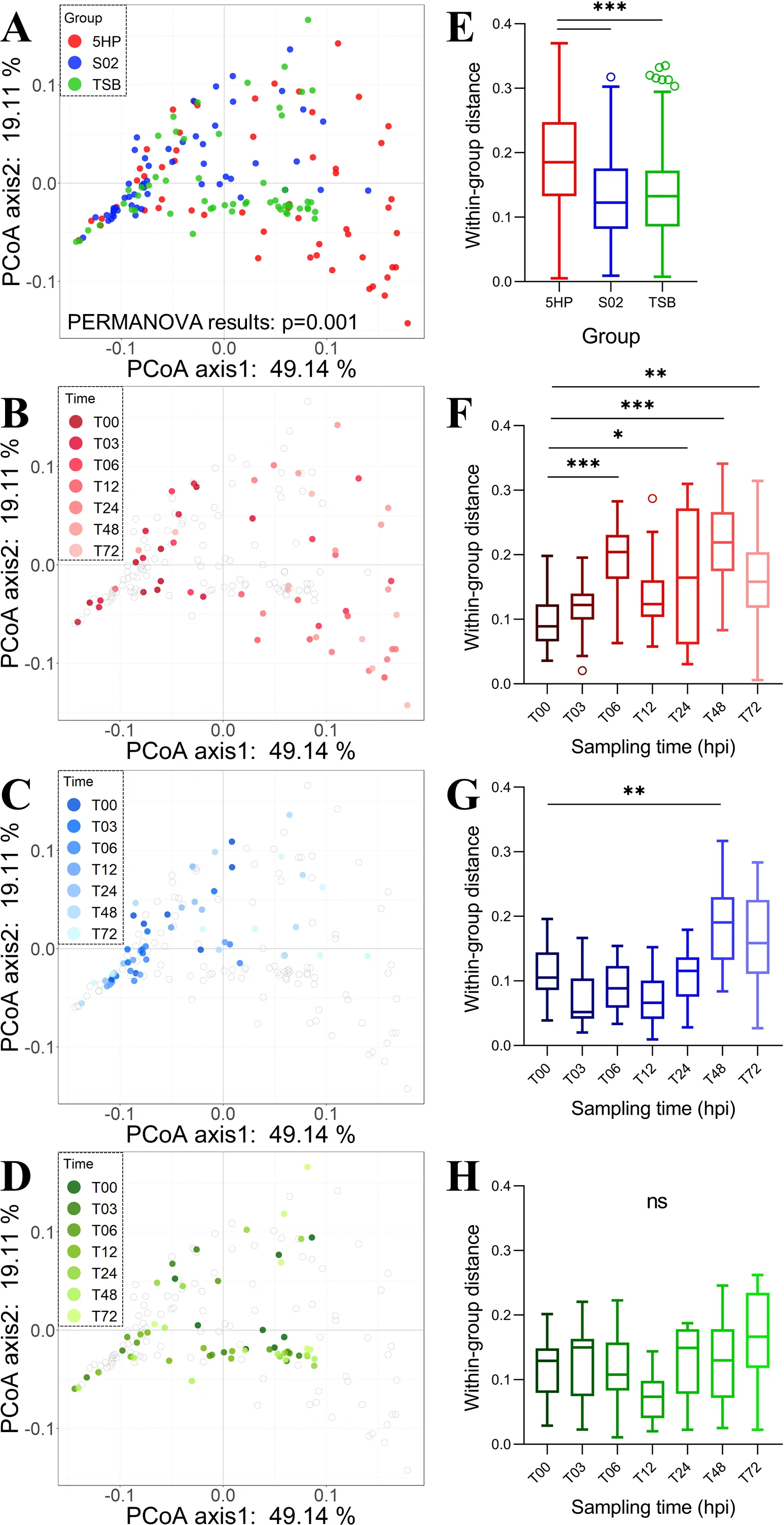
Changes in community composition of gut microbiota among three experimental groups across seven sampling time points. Similarity and dissimilarity in bacterial compositions of gut microbiota were determined by principal co-ordinate analysis (PCoA) based on the weighted UniFrac distances for three experimental groups (5HP-infected, S02-infected, and TSB-treated) or distinct sampling times (T00 to T72) within the group: (**A**) all data points from three groups, (**B**) 5HP-infected group, (**C**) S02-infected group, and (**D**) TSB-treated group. Boxplots (**E–H**) showed the variation of within-group pairwise distances (based on the weighted UniFrac) corresponding to colored groups in panels **A–D**. In PCoA, the numbers of the co-ordinates refer to the variance explained by the axes, and the statistical significance of the differences between the three groups was tested using PERMANOVA test (*P* = 0.001). In boxplots, statistical significance was calculated based on the Kruskal-Wallis test and post hoc Dunn tests. In panels **F–H**, only the statistical results in comparison to T00 are marked. *P < 0.05, **P < 0.01, ***P < 0.001.

Principal co-ordinate analysis (PCoA) based on the weighted UniFrac distances was conducted to illustrate the differences in community composition of gut microbiota (Fig. 4). The permutational multivariate analysis of variance (PERMANOVA) test conﬁrmed the unique compositional characteristics of gut bacterial communities in each group (Fig. 4A; Table S1). In terms of the within-group mean distances, both the S02-infected and TSB-treated groups were significantly lower than the 5HP-infected group, but there was no significant difference in the gut microbiota between S02-infected and TSB-treated groups (Fig. 4E). Specifically, the 5HP-infected group contained samples with relatively high dispersion and exhibited unique taxonomic compositions that differed from the other two groups. Furthermore, while samples in all three groups showed a trend toward dispersion over time (Fig. 4B through D), only the 5HP-infected group had high compositional shifts in the gut microbiota within 6 hours (Fig. 4F through H). Specifically, in the 5HP-infected group, the within-group variation increased significantly from T06 (Fig. 4F), and at T06, the taxonomic composition began to differ significantly from the initial point T00 (Fig. S3A). By contrast, the gut microbiota in the S02-infected group showed higher variation at later T48 and T72 (Fig. 4G; Fig. S3B), and the gut microbiota in the TSB-treated group maintained a constant structure at different time points (Fig. 4H; Fig. S3C).

The random forest analysis confirmed that the gut microbiota of different experimental groups could be well classified and predicted, with an accuracy of 100% and 95.9% for the training (Fig. S4A) and validation (Fig. S4B) data sets, respectively. For the validation data set (Fig. S4B), the 5HP-infected group showed perfect performance in both sensitivity and accuracy (100%), whereas the fitting performance of the S02-infected group was relatively lower with an accuracy of 89.5%, and the sensitivity of the TSB-treated group was relatively lower with an accuracy of 86.7%. These results supported the distinctiveness of the 5HP-infected group and the relative similarity between the S02-infected and TSB-treated groups as observed in the PCoA (Fig. 4A).

### Taxonomic abundance patterns of shrimp gut microbiota

**FIG 5:**
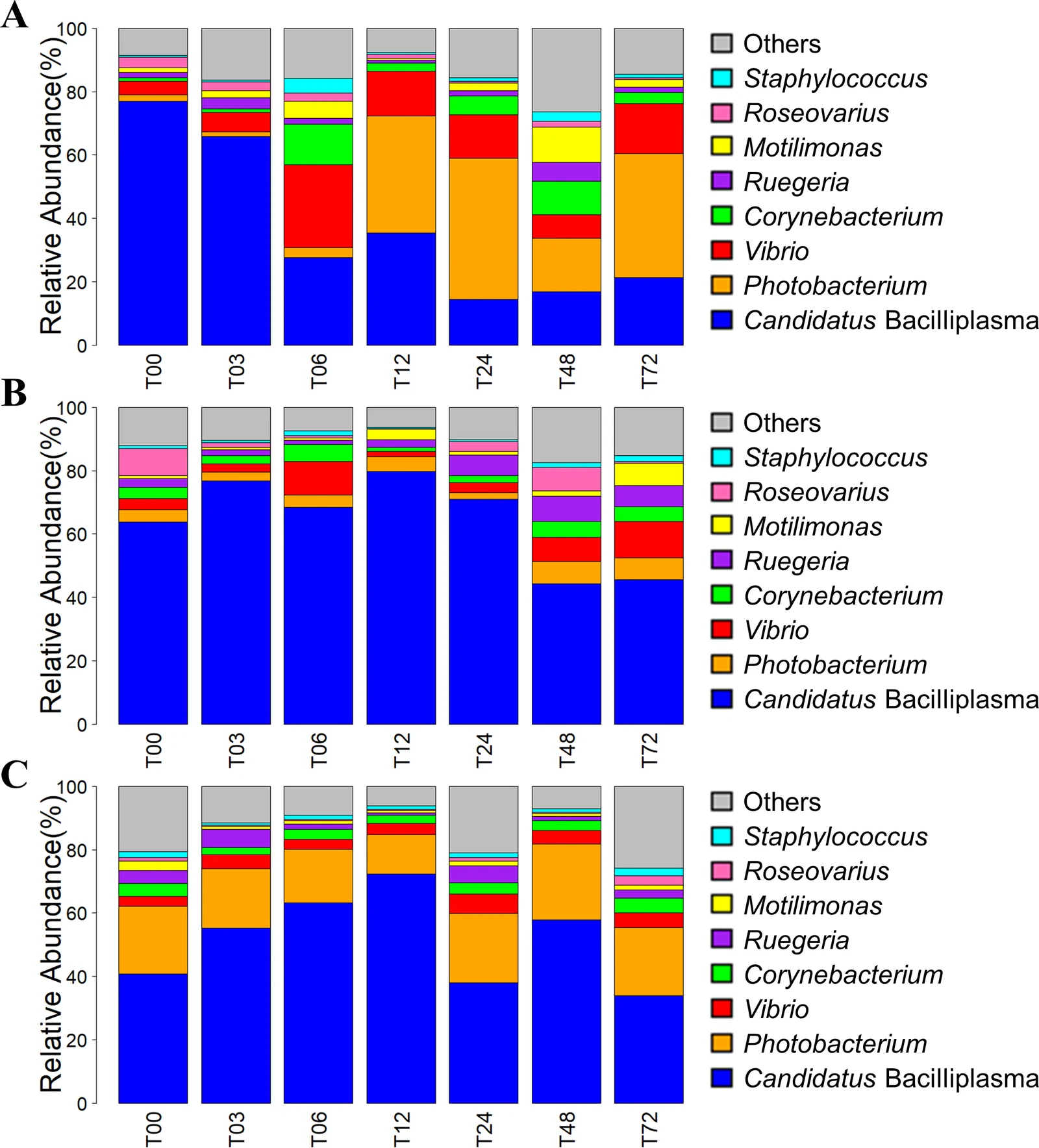
Differences in taxonomic composition of gut microbiota among three experimental groups across seven sampling time points. Bacterial taxonomic composition (at genus level) of shrimp gut microbiota in three experimental groups: (**A**) 5HP-infected, (**B**) S02-infected, and (**C**) TSB-treated groups is shown. Eight dominant genera (with the average abundance greater than 1%) are shown as their relative abundances, while the rest were grouped as “others.”

As revealed by sequence-based taxonomic annotation, eight bacterial genera had an average abundance greater than 1% across all gut samples (Fig. 5). *Candidatus* Bacilliplasma maintained its predominance in the S02-infected and TSB-treated groups at all sampling times (Fig. 5B and C), whereas *Vibrio* and *Photobacterium* were enriched in the 5HP-infected group, with high abundances from T06 to T72 (Fig. 5A).

**FIG 6:**
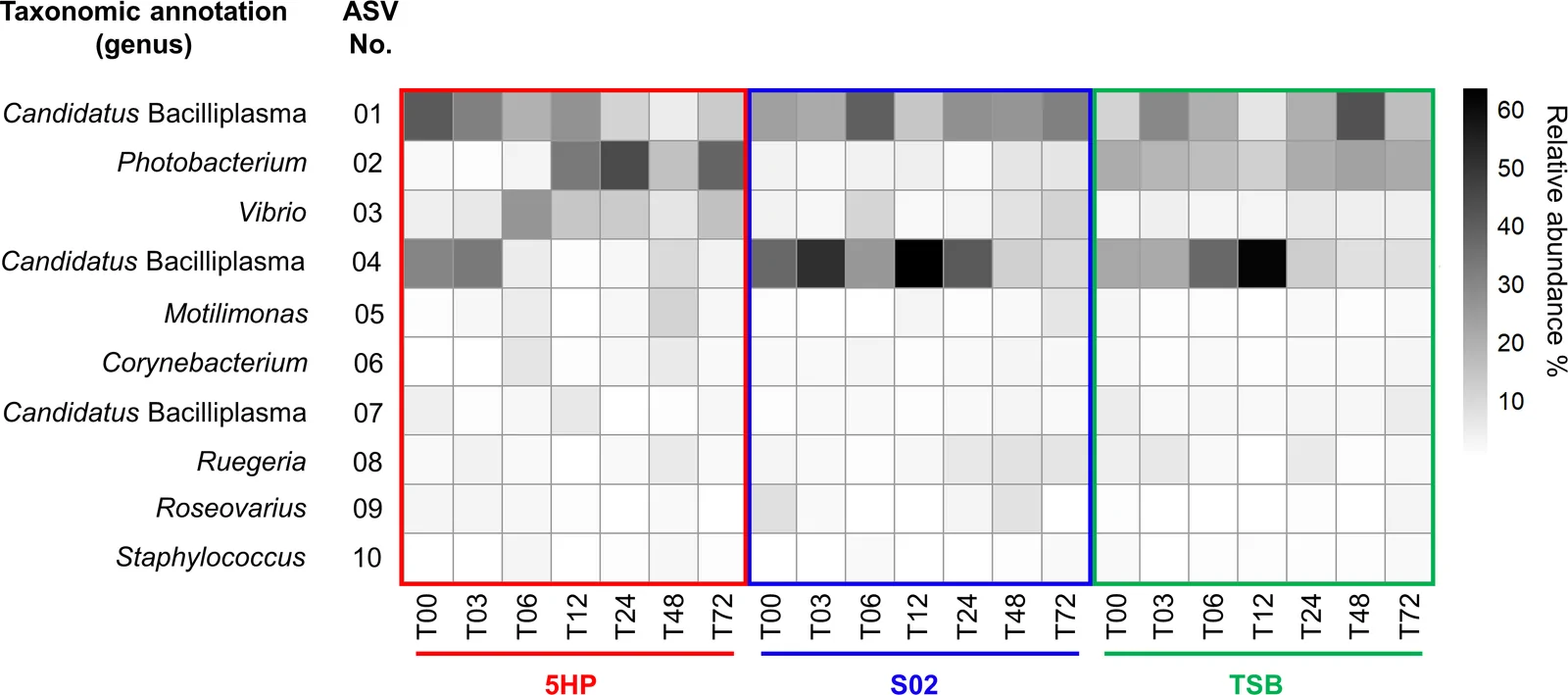
The relative abundances of the top dominant ASVs. Relative abundances of 10 ASVs (with the average abundance greater than 1%) are shown on average for each experimental group at seven time points. The vertical axis shows the ASV number corresponding to the numbers in Tables S5, S6, and S7. The changes in the color intensity indicate the relative abundance of respective ASVs.

Furthermore, we focused on the temporal dynamics of the top 10 dominant ASVs to identify the main contributors to compositional changes over time (Fig. 6). Actually, the top four ASVs cumulatively accounted for ~70% of the total abundance, being good representation. The ASV-01 belonging to C*andidatus* Bacilliplasma existed in a generally rich abundance in all groups and showed weak temporal variation. In contrast, the ASV-04 also belonging to *Candidatus* Bacilliplasma showed a low abundance at T12 in the 5HP-infected group, compared to a high abundance at T12 in the other two groups. The ASV-02 belonging to *Photobacterium* and the ASV-03 belonging to *Vibrio* was particularly increased in the 5HP-infected group, with the highest abundance at T24 and T06, respectively. Importantly, the ASV-03 abundance dynamics might represent the temporal colonization of the *Vibrio* pathogen, as the representative sequence of the ASV-03 was 100% identical to the AHPND-causing Vp 5HP strain.

Through 10-fold cross-validation, the random forest classification identified 33 top-ranking important microbial ASV markers for classifying experimental groups (Fig. S5). Of the top 33 ASVs contributing to the accuracy of the random forest classification model, the dominant ASVs (with average relative abundance >1%, marked as ASV-01 to 10) were all included. These 33 ASVs were mainly from bacterial genera *Candidatus* Bacilliplasma (seven ASVs) and *Corynebacterium* (four ASVs) (Fig. S5).

**FIG 7:**
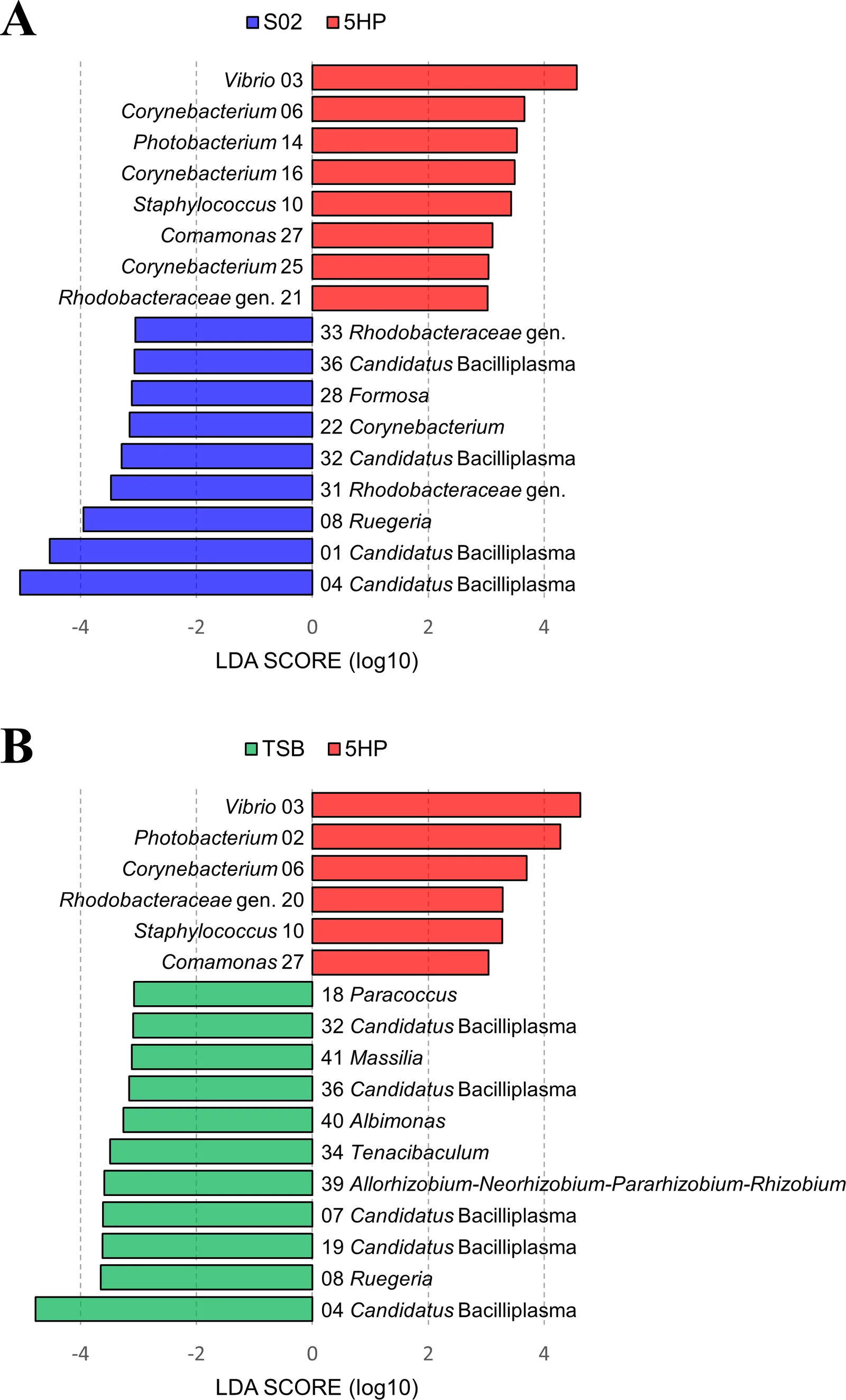
Linear discriminant analysis effect size analysis of the abundance patterns of bacterial ASVs. Identification of bacterial ASVs that differentiated (**A**) 5HP-infected (red) vs S02-infected (blue) groups or (**B**) 5HP-infected (red) vs TSB-treated (green) groups by LDA effect size. The ASV numbers corresponded to the numbers in Tables S5, S6, and S7. The differences were significant (*P*  <  0.05) among classes (Kruskal-Wallis test). The threshold of the logarithmic LDA score was 3.0.

Linear discriminant analysis effect size (LEfSe) analysis revealed that several ASVs showed significant differences in the abundance levels between the 5HP-infected group and the other two groups, respectively [linear discriminant analysis (LDA) >3.0, all *P*  <  0.05; Fig. 7A and B]. Importantly, the top eight ASVs (with mean decrease Gini >1) identified by the random forest analysis (Fig. S5) were also detected by the LEfSe analysis, indicating their high contribution to the discrimination between experimental treatments. *Vibrio* ASV-03 was identified as the most specific feature in the 5HP-infected group with the highest LDA score, while *Candidatus* Bacilliplasma ASV-04 was the most important feature in the S02-infected and TSB-treated groups (Fig. 7A and B). Moreover, ASVs belonging to the genera *Vibrio*, *Photobacterium*, *Staphylococcus*, and *Comamonas* were significantly abundant in the 5HP-infected group, whereas ASVs of the genera *Candidatus* Bacilliplasma and *Ruegeria* were relatively dominant in the S02-infected and TSB-treated groups (Fig. 7).

### Functional analysis of shrimp gut microbiota

**FIG 8:**
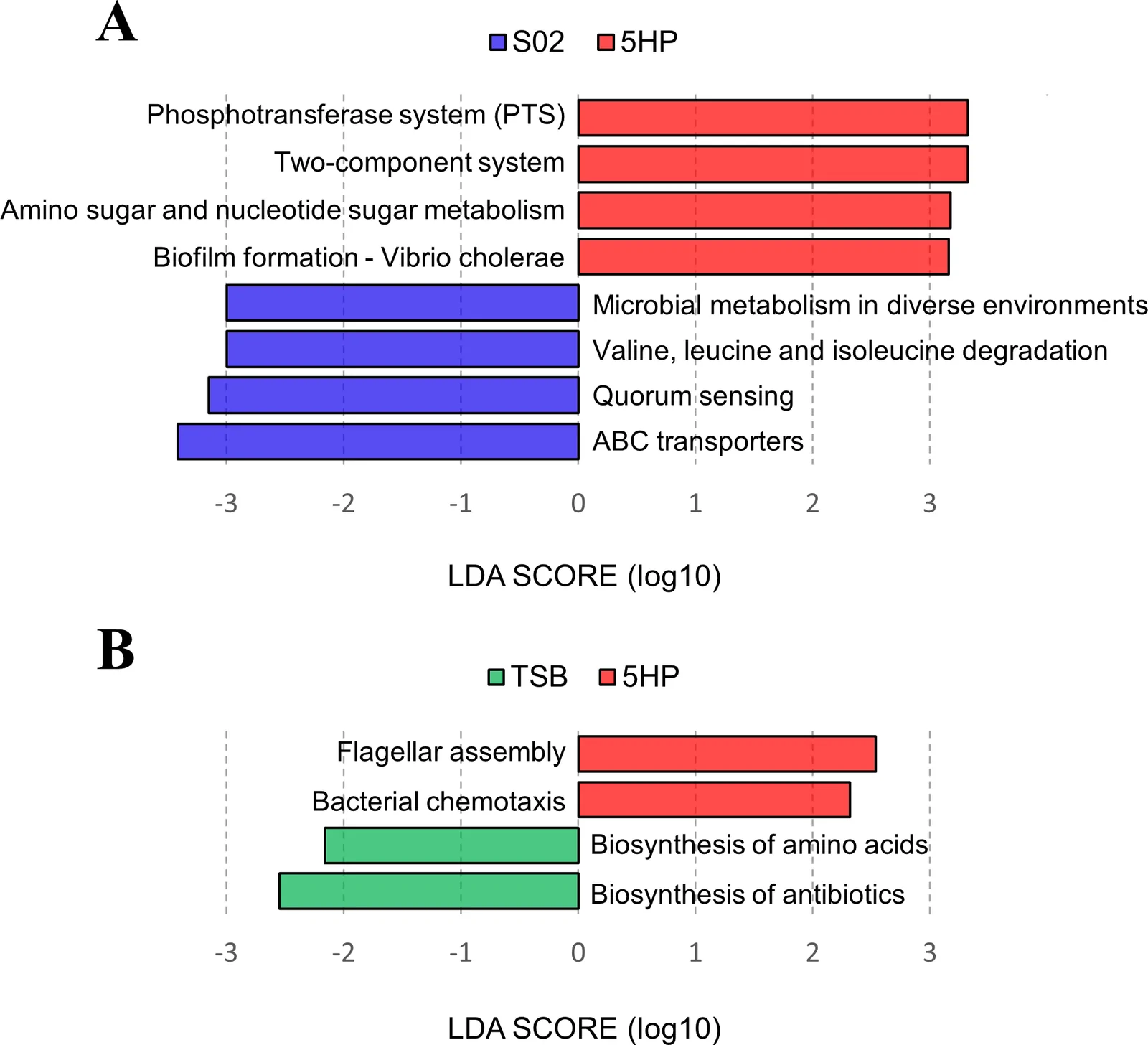
Linear discriminant analysis effect size analysis of functional biomarkers. Identification of functional pathways in KEGG level three that differentiated (**A**) 5HP-infected (red) vs S02-infected (blue) groups or (**B**) 5HP-infected (red) vs TSB-treated (green) groups by LDA effect size. The differences were significant (*P*  <  0.05) among classes (Kruskal-Wallis test). The threshold of the logarithmic LDA score was 3 for panel **A** and 2 for panel **B**.

The potential functions of the shrimp gut microbiota were predicted by Tax4Fun2 through the Kyoto Encyclopedia of Genes and Genomes (KEGG) pathways, and the biomarkers were revealed by LEfSe. Significant differences in eight functional pathways between 5HP-infected and S02-infected groups (LDA >3.0, all *P*  <  0.05) and differences in four functional pathways between 5HP-infected and TSB-treated groups (LDA >2.0, all *P*  <  0.05), respectively, were reported (Fig. 8A and B). In the 5HP-infected group, pathways associated with phosphotransferase system, two-component system, amino sugar and nucleotide sugar metabolism, biofilm formation*—Vibrio cholerae*, flagellar assembly, and bacterial chemotaxis were enriched. In the S02-infected group, functions related to ABC transporters, quorum sensing, valine/leucine/isoleucine degradation, and microbial metabolism in diverse environments were relatively abundant. In the TSB-treated group, pathways associated with biosynthesis of antibiotics and biosynthesis of amino acids were the key features. The highly distinguishable pathways for the 5HP-infected group were mainly associated with signal transduction and cell motility, whereas those for the S02-infected and TSB-treated groups were mainly associated with metabolism (Table S2), suggesting that the shifts in taxonomic compositions of the gut microbiota would likely have an impact on metabolic functions in the gut.

## DISCUSSION

### AHPND-causing Vp colonized the shrimp stomach in a short time

The real-time PCR results showed that the toxin-related genes were apparently detected from 6 to 24 hpi in the 5HP-infected group (Fig. 1), in accord with the enzyme-linked immunoassay conducted by Lai et al. (6). Similar to findings of the previous study (28), the infection of AHPND-causing Vp (the 5HP strain) induced high mortality between 6 and 24 hpi, whereas there was no apparent death caused by the infection of non-AHPND-causing Vp (the S02 strain) (Fig. S1). The detection of toxin-related genes, together with shrimp mortality, suggested that the harmful effect of the AHPND-causing Vp strain correlated with the level of toxin production (29).

However, the sequence abundance of the 5HP strain (annotated as the ASV-03) showed the highest percentage at 6 hpi (Fig. 6) rather than 12 hpi (the high toxin gene detection, Fig. 1). This inconsistency indicated that AHPND-causing Vp might generate plasmids and produce toxins only after stable colonization. Recent evidence suggests that virulence factors of *Vibrio* are regulated by a quorum-sensing system and are expressed only when reaching a certain cell concentration threshold or population density (30, 31), which may explain the time lag between Vp invasion and toxin release. Moreover, these pathogenic processes are consistent with the proposed model of (32) that the incipient entry of AHPND-causing Vp into the shrimp stomach leads to the dysbiosis of microbiota, as a result of direct bacterial competition or the release of signaling metabolites. Moreover, the dysbiosis of the commensal bacterial community would allow the AHPND-causing Vp to further replicate and colonize, with the release of PirAB^Vp^ toxins. Later, the PirAB^Vp^ toxins induce the disruption of tight junctions between stomach epithelial cells, allowing the migration of AHPND-causing Vp to the hepatopancreas. In another study (6), AHPND-causing Vp could be detected in the hepatopancreas at 12 hpi, indicating the later phase of infection (15). Overall, these findings suggest that the colonization resistance of gut microbiota in the early stage (~6 hpi) is critical to prevent or mitigate the effect of AHPND.

### Shrimp gut microbiota diversity reduced after *Vibrio* invasion

In this study, the gut bacterial communities of the *Vibrio*-infected shrimp (5HP-infected and S02-infected groups) showed lower α-diversity values than those of the TSB-treated group, which reflected in species richness (Fig. 2). Specifically, the number of ASVs in the *Vibrio*-infected groups markedly declined for all dominant bacterial genera (Table S3). Species diversity is of great significance for promoting stability and performance in all ecosystems (33, 34), and the diversity of gut microbiota is considered to be a good indicator of host health (35, 36). The disease outbreak is often accompanied by the reduced diversity of microbiota (16, 37, 38); thus, the loss of α-diversity in the 5HP-infected group could be attributed to AHPND. Interestingly, the α-diversity values in the S02-infected group suggested that even exposure to an avirulent strain could reduce the diversity of gut microbiota (Fig. 2). It has been reported that even high concentrations of probiotics may decrease the species richness of shrimp gut microbiota (39). Indeed, exposure to an external microorganism may act as a stressful stimulus which would affect the stability of commensal bacteria (11, 40) as well as alter the relative abundance (41).

Similarly, reduced diversity in the gut microbiota of both *Vibrio*-infected groups (5HP-infected and S02-infected) implicated that a universal initial response of microbiota to opportunistic pathogens is independent of the virulence (42). This initial response indicated that any opportunistic pathogen surrounding the shrimp host may trigger the transition of symbiont gut microbiota and causes a change in health status. Low species diversity in the gut bacterial community would likely become more sensitive to environmental stresses and surrounding microbes (43), reinforcing the emergence of shrimp diseases (44). Our findings, along with previous studies, suggest that the disruption of the symbiotic microbiota is the primary and crucial step in opportunistic pathogen invasion. Thus, developing effective strategies to maintain and restore healthy gut microbiota could be critical for shrimp disease prevention.

### Dysbiosis in shrimp gut microbiota by AHPND-causing Vp

Our study found that bacterial compositions of the gut microbiota were significantly different among healthy (TSB-treated), non-AHPND-infected (S02-infected), and AHPND-infected (5HP-infected) shrimp (Fig. 4). The AHPND-infected (5HP-infected) group was distinct from the healthy (TSB-treated) and non-AHPND-infected (S02-infected) groups, as evidenced by within-group β-distances (Fig. 4E) and random forest analysis (Fig. S4). This result is in accordance with previous report (17) where AHPND-infected shrimp showed microbiota compositions that were distinct from healthy shrimp. Our findings indicate that AHPND-causing Vp adapts unique strategies to break the colonization resistance of commensal bacteria and obtain a competitive advantage. Compared to non-AHPND-causing Vp, the presence of a pVA plasmid carrying PirAB^Vp^ toxin genes along with other virulence genes (45) in the AHPND-causing Vp may serve as a characteristic feature to outcompete other microbes. In addition to the plasmid, an AHPND-causing Vp strain (13–028/A3 strain) has been reported to utilize multiple carbon sources more efficiently than non-AHPND-causing Vp (RIMD2210633 and BB22OP strains) (27). The capability to efficiently metabolize various substrates contributes to the competitive advantage of microbes (46).

Considering the temporal changes in gut microbiota, in the 5HP-infected group, the gut microbiota composition shifted and the variation increased at 6 hpi, while the microbiota in the S02-infected and TSB-treated groups maintained a relatively stable structure during the course of the experiment (Fig. 4). The minor fluctuations of microbiota in the TSB-treated group is representative of usual state in a healthy host (47 – 49). The exposure to both AHPND-causing Vp and non-AHPND-causing Vp, respectively, could perturb the community composition. However, a resilient community could recover to normal functions after a lag phase (49). The recovery process in the S02-infected group was reflected during 6 to 24 hpi by the increasing abundance of ASVs that belong to *Candidatus* Bacilliplasma and maintained diverse genus-level composition during 48 to 72 hpi (Fig. 5). However, in the 5HP-infected group, the perturbation by the AHPND-causing Vp would directly change the taxonomic composition of gut microbiota, exceeding the threshold of resilience.

The relative abundances of *Vibrio* and *Photobacterium* were increased in the gut microbiota of the 5HP-infected shrimp, while *Candidatus* Bacilliplasma maintained its predominance in the S02-infected and TSB-treated shrimp (Fig. 5). Both *Vibrio* and *Photobacterium* belong to the Vibrionaceae family. Vibrionaceae has served as the signature for AHPND diagnosis (50). The higher abundances of the Vibrionaceae family and *Vibrio* genus were postulated to be caused by the colonization of the AHPND-causing Vp and related to secondary *Vibrio* infections (51). Apart from some highly virulent strains causing the primary disease, *Vibrio* spp. are often considered as secondary or opportunistic pathogens in shrimp (52). Most shrimp vibrioses occur either combined with physical stresses or following primary infections by other pathogens (53). An argument is that AHPND is not a typical vibriosis infection but an acute intoxication caused by PirAB^Vp^ toxins (8). However, if PirAB^Vp^ toxins could modulate the virulence of non-AHPND-causing *Vibrio* species (9), shrimp may indeed die because of a secondary vibriosis after being weakened by PirAB^Vp^ toxins. In addition, the relatively high abundance of *Photobacterium* may be associated with secondary luminous bacterial infections (54), as reported in pearl gentian grouper, where infection with high virulent *Vibrio* significantly increased the abundance of predominant *Photobacterium* (55). It has been suggested that the stress from the invasion of *Vibrio* might open a niche for *Photobacterium* to opportunistically occupy (56).

*Candidatus* Bacilliplasma, reported as a possible novel lineage of class *Mollicutes* (57), is often observed in the digestive tract of shrimp and has been implicated in several shrimp diseases (42, 58, 59). As a prominence in the shrimp gut, the abundance of *Candidatus* Bacilliplasma has been reported to decrease in shrimp subjected to microcystin-LR (a variant of microcystin with leucine and arginine) stress and White spot syndrome virus (WSSV) infection (60, 61). In our study, the ASV-04 belonging to *Candidatus* Bacilliplasma exhibited an inverse trend to ASV-03 belonging to *Vibrio* (Fig. 7), which indicated its antagonism to the opportunistic or pathogenic bacterial strains. Our finding implied that the decrease of *Candidatus* Bacilliplasma may indicate the diseased status of shrimp. However, by analyzing bacterial interaction networks, Chen et al. (42) suggested that varied subspecies of *Candidatus* Bacilliplasma interacted with the pathogenic *Vibrio* strains, and they either enhance or inhibit infection. Thus, the interaction dynamics between ASVs belonging to *Candidatus* Bacilliplasma and *Vibrio* should be reassessed further to understand their interaction mechanisms.

### Microbiota regulates the maintenance of host gut functions

The functional pathways of shrimp gut microbiota infected by AHPND-causing Vp (5HP-infected) were mainly associated with “signal transduction” and “cell motility,” while those in non-AHPND-causing Vp (S02-infected) and control (TSB-treated) groups were mainly associated with “metabolism” (Table S2). In diseased shrimp, pathogens are extremely efficient in arranging their virulence repertoire through complex signaling transduction systems in response to the compound signals from the host or the normal gut microbiota (62). The distinguished pathways of cell motility were supposed to be enriched due to the dual flagellar systems of Vp strains adapted for locomotion under different circumstances (63). In healthy shrimp, the gut microbiota provides baseline functions associated with general metabolism, especially in the metabolism of amino acids and carbohydrates (64). It is generally considered that aquatic animals do not have all the essential enzymes to cope with their dietary challenges (65). For example, low activities of polysaccharide digestive enzymes were detected in the intestine of shrimp (66). Several bacterial strains isolated from the shrimp digestive tract have the genetic capacity to produce extracellular enzymes (67). Thus, the microbiota producing digestive enzymes was inferred to remedy the shortage (64). Specifically, the gut microbiota plays a major role in protein digestion, amino acid metabolism, lipid metabolism, and fatty acid synthesis that are vital for host health (68, 69).

Kumar et al. (70) previously suggested that the induction of genes involved in bile acid synthesis was observed in response to AHPND. Crude bile acids positively influence both biofilm formation and the secretion of PirAB^Vp^ toxins (71). Elevated taurocholate concentrations were correlated with enhanced biofilm formation in AHPND-causing Vp (70), consistent with the results from our LEfSe analysis that the biofilm formation was enriched in the AHPND-causing Vp (5HP-infected) group (Fig. 8). However, bile acid and taurocholic bile salt treatments failed to induce the biofilm formation of the non-AHPND-causing Vp (S02), which may be due to the significant genomic differences between the AHPND-causing and non-AHPND-causing strains (4). Pathogens may resist the antibacterial properties of bile and may also use bile as a signal to modulate their virulence (72). Thus, the effects of bile acid constituents on pathogen biofilm formation are selective and distinct (73). The differential utilization of metabolites results in the differential effects of pathogenic and non-pathogenic bacterial invasions on the gut microbiota.

Our results, together with previous studies, indicate that the gut microbiota plays an indispensable role in host metabolic functions, highlighting the importance of maintaining a balance in the shrimp gut microbiota. We found that AHPND-causing Vp could induce dysbiosis of the shrimp microbiota, altering the metabolic functions of gut bacteria. In contrast, non-AHPND Vp was found to induce non-pathological changes in the gut microbiota, suggesting that the gut microbiota responds differently to pathogenic and non-pathogenic *Vibrio parahaemolyticus*.

## MATERIALS AND METHODS

### Experimental animals and bacterial strains

Specific pathogen-free Pacific white shrimp (*Litopenaeus vannamei*, with average body weight 2.0 ± 0.5 g) were obtained from the National Pingtung University of Science and Technology. Shrimp were kept in tanks containing 30 L of artificial seawater (maintaining salinity ~20 ppt, temperature ~27°C, and pH value ~8.0) for 2 days as acclimatization prior to the immersion challenge. During the experimental periods, environmental conditions of the water tanks were kept the same as described above for all groups (Table S4).

Two strains of Vp were used for this study: 5HP strain (AHPND-causing Vp) and S02 strain (non-AHPND-causing Vp) both isolated from Thailand (74). Bacterial glycerol stocks were provided by the laboratory of Prof. Han-Ching Wang in the National Cheng Kung University and preserved in 25% glycerol at −80℃ before the immersion challenge.

### Experimental groups and immersion challenges

To explore the Vp infection effects on the gut microbiota, we performed three experimental treatments with (i) 5HP-infected group: immersion challenge with Vp 5HP strain, (ii) S02-infected group: immersion challenge with Vp S02 strain, and (iii) TSB-treated group: immersion in the TSB as a negative control. To detect the change in gut microbiota during the critical period of AHPND pathogenesis (32), shrimp stomach samples were collected, respectively, at seven time points (0, 3, 6, 12, 24, 48, and 72 hours post immersion; shown as T00, T03, T06, T12, T24, T48, and T72 hereafter).

Shrimp were randomly distributed into six tanks (three treatments in duplicates, *n* = 35/per tank). The immersion challenges were performed as described previously by Lai et al. (6) with slight modifications. To recover bacterial stocks, Vp 5HP and S02 strains were separately cultured on thiosulfate citrate bile salts sucrose agar plates. The colonies were inoculated into TSB medium with 2% NaCl and incubated at 30°C, 180 rpm, for 16 hours as starting cultures. Overnight bacterial culture was then scaled up and the cell density was adjusted to OD_600_ = 0.1 (approximately 10^7^ CFU/mL) assessed by spectrophotometer. Individual bacterial inoculums (100 mL) were mixed with 900-mL seawater to adjust the concentration to 10^6^ CFU/mL for immersion challenges. For the 5HP-infected and S02-infected groups, shrimp were immersed in the 10^6^-CFU/mL inoculum mixture for 15 min and then transferred back to their respective tanks. To keep shrimp under the infected condition, ~300-mL inoculum mixture was added to 30-L seawater in the tank (final bacterial density 10^4^ CFU/mL). At T00, T03, T06, T12, T24, T48, and T72, the entire stomach of each shrimp, including the contents and mucosa, was aseptically dissected and stored at −80°C until DNA extraction. The stomach was sliced into two parts: one-fifth of the stomach was used for AHPND diagnosis and the rest was kept for gut microbiome analysis (42). At each time point, four shrimp individuals were collected from each tank, giving a total of 168 samples for subsequent data analysis.

### AHPND detection

The DNA for AHPND diagnosis was extracted from shrimp stomach using a DTAB/CTAB DNA extraction kit (GeneReach Biotechnology Corp, Taiwan). The DNA yield and quality were assessed by a NanoDrop spectrophotometer (Thermo Fisher Scientific, USA). To quantitatively determine AHPND-related markers, TaqMan real-time PCR was performed on a CFX96 real-time system (Bio-Rad, USA), using an IQ REAL AHPND/EMS Quantitative System (targeting the AHPND plasmid and PirAB^Vp^ gene) and an IQ REAL WSSV Quantitative System (targeting host genome) (Gene Reach Biotechnology Corp, Taiwan). A two-temperature PCR amplification protocol was applied: 40 cycles of denaturation at 93°C for 15 s and annealing/extension at 60°C for 1 min (42). The artificial DNA provided in the kit contained partial sequence fragments of the AHPND plasmid and PirAB^Vp^ gene (Toxin 1), which were used as standards to construct standard curves. The relative copy number of the AHPND plasmid or toxin 1 was normalized per cell against host genome copies. One-way analysis of variance with Dunnett’s test was performed using the GraphPad Prism V.8 software for Windows (GraphPad Software, USA; https://www.graphpad.com/) to evaluate the differences in the copy numbers of AHPND-associated genes over time.

### Microbiome profiling by 16S rRNA gene sequencing

The DNA for gut microbiome analysis was extracted using QIAamp PowerFecal DNA Kit (QIAGEN, German), according to the manufacturer’s instructions. The hypervariable V4 region of the 16S rRNA gene was amplified by the specific 515F/806R PCR primers (515F: GTGYCAGCMGCCGCGGTAA, 806R: GGACTACNVGGGTWTCTAAT) (75), following the steps: initial denaturing at 95°C for 3 min; 28 cycles of 95°C for 30 s, 55°C for 40 s, 72°C for 50 s; and a final extension at 72°C for 5 min. The sizes of PCR products were checked by gel electrophoresis. For the samples with only one specific band, the PCR products were purified by Agencourt AMPuer XP (Beckman Coulter, USA), while those with multiple bands were purified by QIAquick Gel Extraction Kit (QIAGEN) focusing on the band with the expected size. To build the amplicon library for high-throughput sequencing, the PCR products were bound to adapters with barcodes as follows: initial denaturing at 95°C for 3 min; 7 cycles of 95°C for 30 s, 55°C for 40 s, 72°C for 50 s; and a final extension at 72°C for 5 min. After confirming the product size and purification, the amplicon concentrations were measured by Qubit 1X dsDNA HS Assay Kit on a QubitTM Fluorometer. Pooled library containing equal DNA concentration for each sample was sequenced on the Illumina Miseq platform, with 2 × 300 bp paired-end reads (Genomics BioSci. & Tech, Taiwan).

### Processing of raw sequencing data

Using QIIME2 v.2021.11 (76), paired-end FASTQ sequence reads were demultiplexed based on sample unique barcodes. The PCR primer flanks were trimmed using Cutadapt (77). Quality filtration, sequence merging, and feature abundance table were conducted by the DADA2 plugin (78). Sequence bases with low Q scores were truncated considering Q30 as the benchmark. The truncated reads were further grouped into ASVs, followed by chimera removal. The representative sequences were extracted to generate a rooted phylogenetic tree, produced by the QIIME2 phylogeny tool: align-to-tree-mafft-fast tree (79, 80). ASVs were taxonomically classified from phylum to genus levels by using the classify-sklearn with a naïve Bayes classifier trained on Silva 138 99% operational taxonomic units (OTUs) from the 515F/806R region of sequences (MD5: e05afad0fe87542704be96ff483824d4) (40, 81 – 83). ASVs that were not assigned to bacteria (i.e., unassigned, archaeal, and eukaryotic ASVs, as well as chloroplast and mitochondrial ASVs) were excluded.

The feature abundance table generated by DADA2 was rarefied to a depth of 8,800 sequence reads per sample. For equal comparison, two samples of the S02-infected group and one sample of the TSB-treated group were excluded due to insufficient reads. The final rarefied table contained a total of 165 samples.

### Microbiome community analysis

To detect the changes in shrimp gut microbiota, α-diversity indices, including observed features, Chao1, and Shannon were estimated using QIIME2 (76). To determine the differences in bacterial community composition, PCoA based on weighted UniFrac distances were applied to analyze and visualize the patterns of β-diversity (84, 85). The diversity core-metrics-phylogenetic plugin in QIIME2 was used to calculate the weighted UniFrac distances, and the β-diversity was visualized through PCoA using the ggplot function from ggplot2 package (86) in R v.4.2.0 (87). Boxplots of within-group α-diversity and β-diversity were performed using the GraphPad Prism v.8 software for Windows (GraphPad Software, https://www.graphpad.com/). Kruskal-Wallis tests and post hoc Dunn tests were performed using the GraphPad Prism v.8 software for Windows (GraphPad Software, https://www.graphpad.com/) to assess differences in α-diversity values, within-group β-diversity distances, and temporal compositional variations among three experimental groups, with the significance level set at *P* < 0.05. Moreover, PERMANOVA was employed using QIIME2 (76) to evaluate differences in the gut microbiota among three experimental groups, with the significance level set at *P* < 0.05.

To test whether the gut microbiota of different experimental groups could be well classified and predicted, a random forest model was constructed with the abundances of ASVs, using package randomForest in R (88). The samples were randomized into a training data set (70%) and a validation data set (30%). The contribution of ASVs to group classification was based on the mean decrease of Gini. To identify the top-ranking important ASVs, the predictive performance was evaluated using 10-fold cross-validation with the random forest model.

To further infer the functional potential of the gut microbiota, Tax4Fun2 v.1.1.5 (89) based on the KEGG database (90) was applied for acquiring the functional composition of the gut microbiota, and the predicted functional categories of Tax4Fun2 were grouped into three levels according to the KEGG database.

To identify the taxonomic or functional biomarkers that differed significantly between experimental treatments, the LEfSe (91) was applied for biomarker discovery using the Galaxy/Hutlab tool (https://huttenhower.sph.harvard.edu/galaxy/). The significance level was set at *P* < 0.05 and the threshold for LDA scores was 3.0.

## Data Availability

The raw sequencing data reported in this study has been archived in the Sequence Read Archive of the National Center for Biotechnology Information under accession number PRJNA921960.

## SUPPLEMENTAL MATERIAL

Supplemental figures and tables spectrum.01180-23-s0001.docx Fig. S1 to S5 and Tables S1 to S7.

ASM does not own the copyrights to Supplemental Material that may be linked to, or accessed through, an article. The authors have granted ASM a non-exclusive, world-wide license to publish the Supplemental Material files. Please contact the corresponding author directly for reuse.
