# Supplementary material for: Gut microbiota of Pacific white shrimp (Litopenaeus vannamei) exhibits distinct responses to pathogenic and non-pathogenic Vibrio parahaemolyticus
Source: Microbiol Spectr. 2023 Sep 26;11(5):e01180-23. doi: 10.1128/spectrum.01180-23 (PMC10580984; doi:10.1128/spectrum.01180-23)
Supplement: Supplemental figures and tables — Fig. S1 to S5 and Tables S1 to S7. [file spectrum.01180-23-s0001.docx]

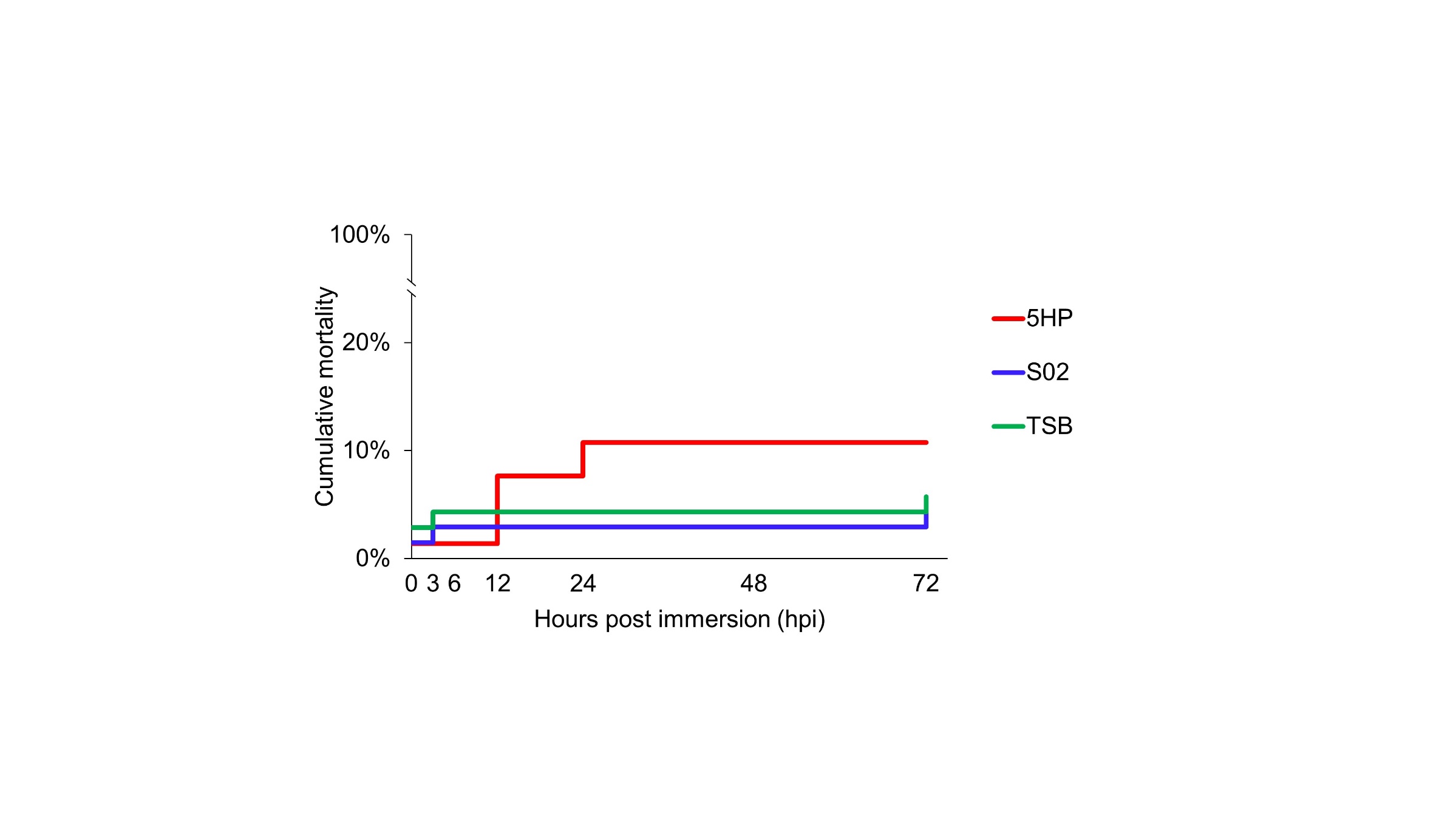


**Fig. S1 Cumulative mortality of shrimp at 72 hpi for three experimental groups.** The data show mean values of cumulative mortality over time after the immersion challenge. Different colors of lines indicate samples from three distinct experimental groups: 5HP = red, S02 = blue, and TSB = green.


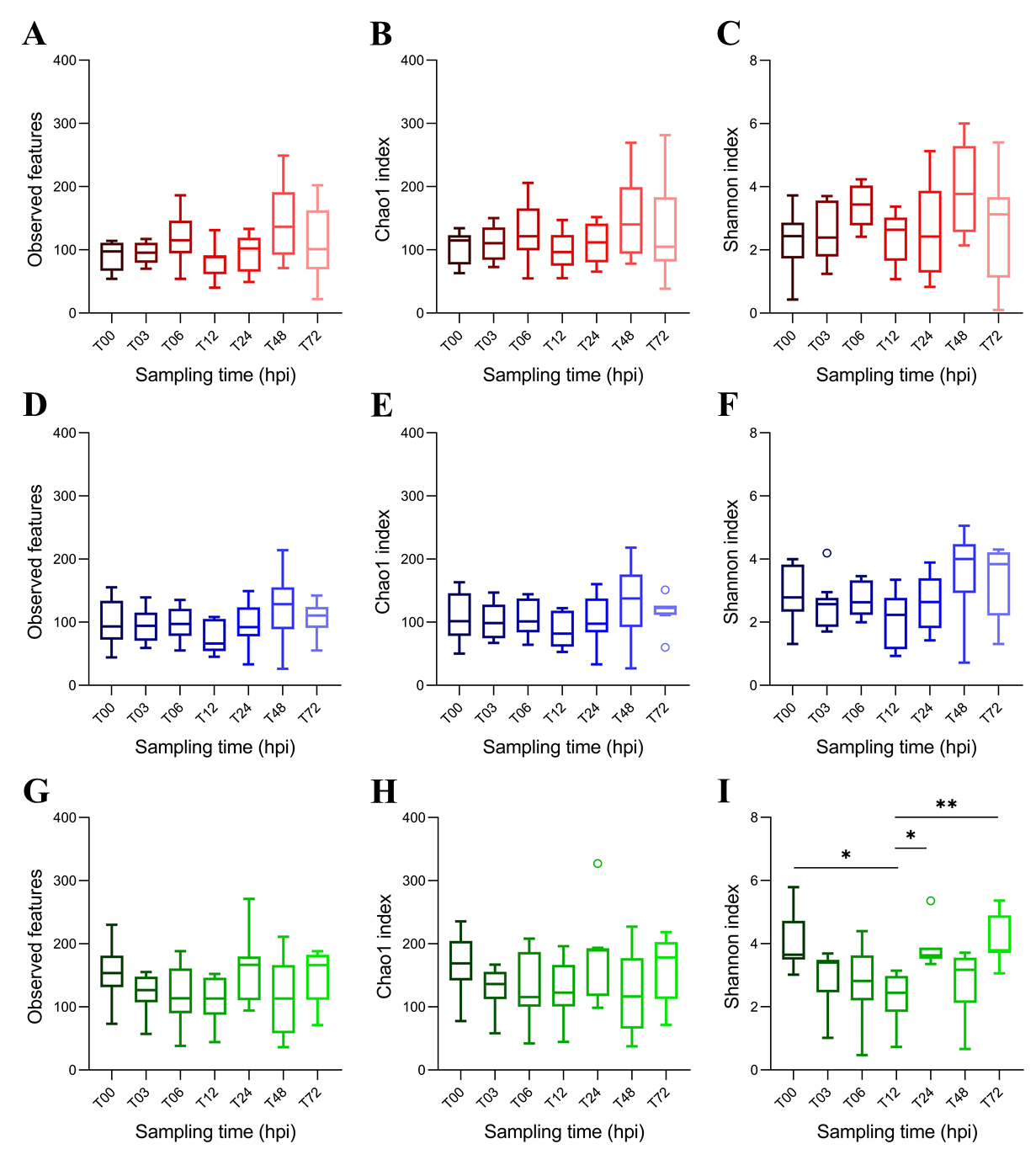


**Fig. S2 Comparison of microbial α-diversity at different time points for three experimental groups.** Microbial α-diversity estimated by distinct diversity indices (including observed features, Chao1 index, and Shannon index) are shown at different time points in three experimental groups: (A-C) 5HP, (D-F) S02, and (G-I) TSB. Different colors indicate samples from different groups and shades were used to distinguish T00 to T72 time points. Statistical significance was calculated based on the Kruskal-Wallis test and post-hoc Dunn tests. **: *p* < 0.01, *: *p* < 0.05.


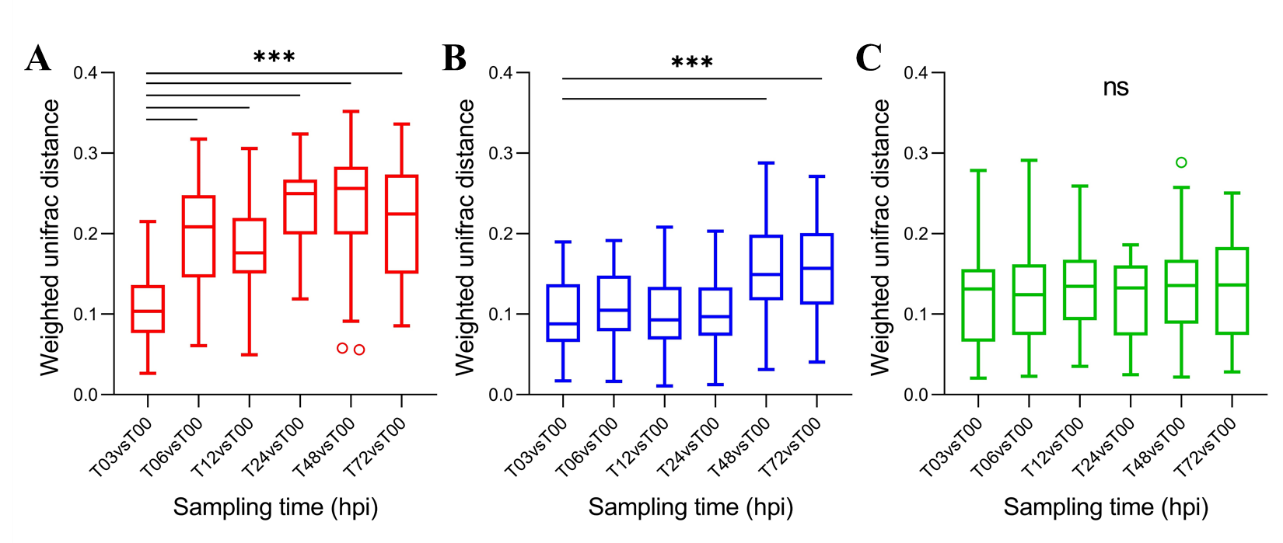


**Fig. S3 Compositional changes of gut microbiota at different time points.** The changes in composition are compared with the start point T00, revealed by between-group weighted UniFrac distances. Distinct patterns were detected for samples in (A) 5HP, (B) S02 and (C) TSB experimental groups. Statistical significance was calculated based on the Kruskal-Wallis test and post-hoc Dunn tests. Only the statistically results comparing to T00 were marked. ***: p < 0.001.


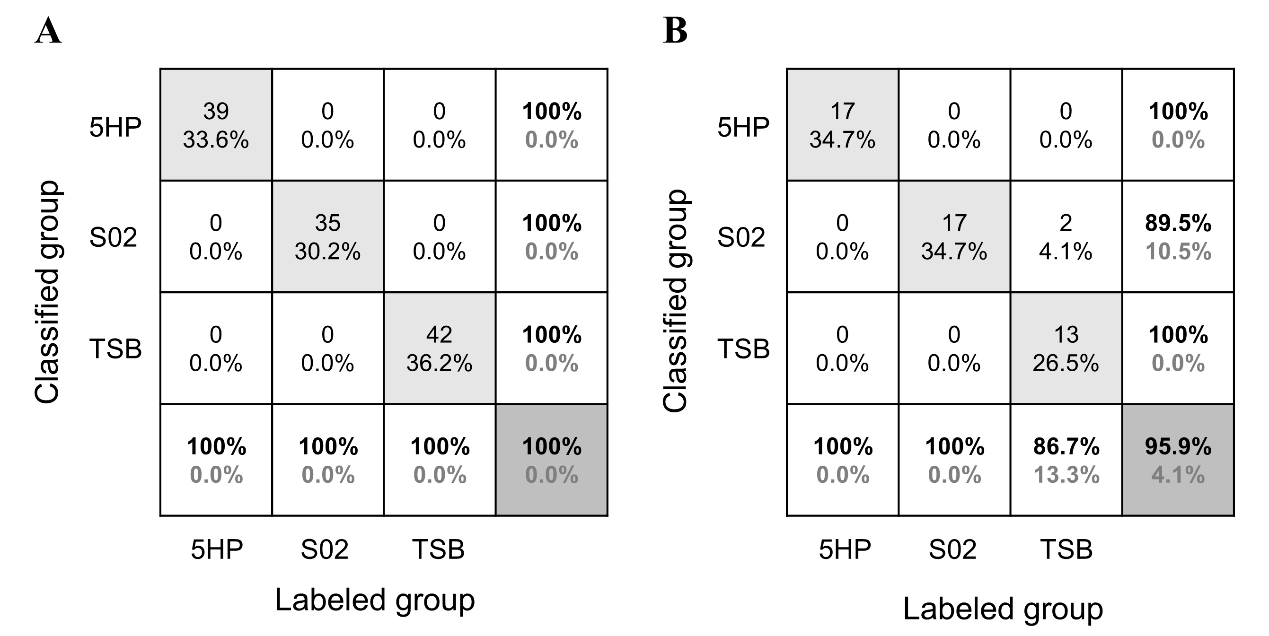


**Fig. S4 Confusion matrices of the training and validation datasets in the random forest predictions.** As shown by the lower right element of the matrix reports, the overall accuracy for the (A) training and (B) validation datasets in the random forest was 100% and 95.9%, respectively. The sensitivity for each group is shown in the bottom row, and the precision is shown in the rightmost column. The diagonal shows the true positive values for each group (labeled and classified as that group), while the numbers off the diagonal report any confusion between the classes. When the fitted classification model is used to re-identify the training dataset, it even corrects the misclassifications made during the fitting process, with an accuracy of 100%. For the validation dataset, the 5HP-infected group shows perfect performance in both sensitivity and accuracy, while the fitting performance of the S02-infected group is relatively lower, with an accuracy of 89.5%, and the sensitivity of the TSB-treated group is relatively lower, with an accuracy of 86.7%.


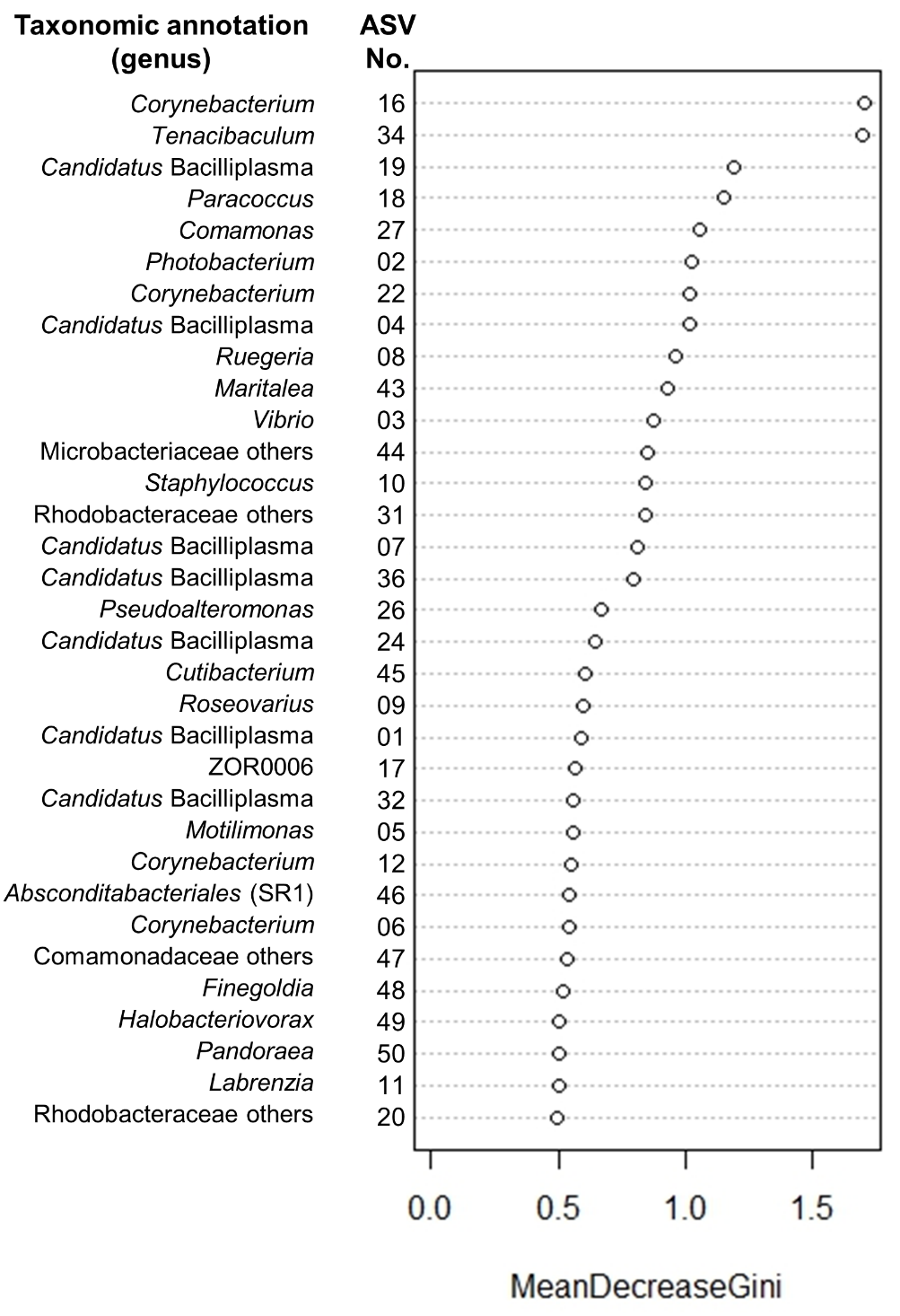


**Fig. S5 Random forest (RF) classification for gut microbiota among three experimental groups.** A ten-fold cross-validation was performed and as a result, 33 ASV markers were selected as the optimal marker set. The ASVs are ranked according to the mean decrease of Gini in classifying the data. Of the top 33 ASVs contributing to the accuracy of the random forest classification model, the dominant ASVs (with average relative abundance > 1%, marked as ASV No. 01 to 10) were all included.

**Table S1. Pairwise PERMANOVA results comparing shrimp gut microbiota** **composition across three pairs of experimental groups.**

| **Pair** | **Sample size** | **Permutations** | **pseudo-F** | **p-value** | **q-value** |
| --- | --- | --- | --- | --- | --- |
| 5HP vs S02 | 110 | 999 | 14.27578567 | 0.001 | 0.003 |
| 5HP vs TSB | 111 | 999 | 4.597072656 | 0.005 | 0.005 |
| S02 vs TSB | 109 | 999 | 5.555687042 | 0.002 | 0.003 |

**Table S2. Functional biomarkers revealed by LEfSe belonging to three levels of KEGG pathways.**

| **Level 1** | **Level 2** | **Level 3** | **Biomarkers (treatment groups)** |
| --- | --- | --- | --- |
| Cellular Processes | Cell motility | Flagellar assembly | 5HP |
|  |  | Bacterial chemotaxis | 5HP |
|  | Cellular community - prokaryotes | Biofilm formation - *Vibrio cholerae* | 5HP |
|  |  | Quorum sensing | S02 |
| Environmental Information Processing | Membrane transport | Phosphotransferase system (PTS) | 5HP |
|  |  | ABC transporters | S02 |
|  | Signal transduction | Two-component system | 5HP |
| Metabolism | Amino acid metabolism | Valine, leucine and isoleucine degradation | S02 |
|  | Carbohydrate metabolism | Amino sugar and nucleotide sugar metabolism | 5HP |
|  | Global and overview maps | Microbial metabolism in diverse environments | S02 |
|  |  | Biosynthesis of amino acids | TSB |
|  |  | Biosynthesis of antibiotics | TSB |

**Table S3. The number of ASVs in the abundant genera among 3 experimental groups.**

|  | 5HP | S02 | TSB |
| --- | --- | --- | --- |
| *Candidatus* Bacilliplasma | 15 | 80 | 238 |
| *Photobacterium* | 6 | 26 | 114 |
| *Vibrio* | 21 | 18 | 107 |
| *Corynebacterium* | 19 | 20 | 54 |
| *Ruegeria* | 4 | 4 | 13 |
| *Rhodobacteraceae* gen. | 67 | 76 | 147 |
| *Motilimonas* | 1 | 5 | 33 |
| *Roseovarius* | 2 | 2 | 4 |
| *Staphylococcus* | 5 | 7 | 26 |
| Total ASVs | 1166 | 1354 | 2868 |

**Table S4. Environmental measurements of three experimental groups at each time point.**

|  | **Factor** | **Temperature (℃)** | | | | | | | | |  | **pH** | | | | | | | | |  |
| --- | --- | --- | --- | --- | --- | --- | --- | --- | --- | --- | --- | --- | --- | --- | --- | --- | --- | --- | --- | --- | --- |
|  | **Treatment** | **5HP infected** | | | **S02 infected** | | | **TSB treated** | |  |  | **5HP infected** | | | **S02 infected** | | | **TSB treated** | |  | |
| **Time**  **point** | **Tank No.** | **5HP-1** | **5HP-2** | **S02-1** | | **S02-2** | **TSB-1** | | **TSB-2** | **Average**  **± std** |  | **5HP-1** | **5HP-2** | **S02-1** | | **S02-2** | **TSB-1** | | **TSB-2** | **Average**  **± std** | |
| T00 |  | 27.0 | 27.0 | 27.0 | | 27.0 | 26.5 | | 27.0 | 26.9±0.2 |  | 7.8 | 7.7 | 7.6 | | 7.7 | 7.8 | | 7.7 | 7.7±0.1 | |
| T03 |  | 27.0 | 27.0 | 27.0 | | 27.0 | 27.0 | | 27.0 | 27.0±0.0 |  | 7.5 | 7.7 | 7.5 | | 7.7 | 7.8 | | 7.7 | 7.7±0.1 | |
| T06 |  | 27.0 | 27.0 | 27.0 | | 27.0 | 27.0 | | 27.0 | 27.0±0.0 |  | 7.4 | 7.6 | 7.4 | | 7.7 | 7.7 | | 7.7 | 7.6±0.2 | |
| T12 |  | 27.0 | 27.0 | 27.0 | | 27.0 | 27.0 | | 27.0 | 27.0±0.0 |  | 7.4 | 7.7 | 7.6 | | 7.8 | 7.8 | | 7.8 | 7.7±0.2 | |
| T24 |  | 27.0 | 27.0 | 27.0 | | 27.0 | 27.0 | | 27.0 | 27.0±0.0 |  | 7.9 | 7.9 | 7.8 | | 8.0 | 8.0 | | 7.9 | 7.9±0.1 | |
| T48 |  | 27.0 | 27.0 | 27.0 | | 27.0 | 27.0 | | 27.0 | 27.0±0.0 |  | 7.7 | 7.9 | 7.9 | | 8.1 | 8.1 | | 8.0 | 7.9±0.2 | |
| T72 |  | 27.0 | 27.0 | 27.0 | | 27.0 | 27.0 | | 27.0 | 27.0±0.0 |  | 7.8 | 8.0 | 8.1 | | 8.1 | 8.2 | | 8.0 | 8.0±0.1 | |

**Table S5. Annotation of the top 30 ASVs in shrimp gut microbiota of the 5HP-infected group.**

| ASV  No. | Average abundance (%) | Taxonomic annotation (phylum to genus) |
| --- | --- | --- |
| 01 | 21.26 | p__*Firmicutes*; c__*Bacilli*; o__*Mycoplasmatales*; f__*Mycoplasmataceae*; g__*Candidatus* Bacilliplasma |
| 02 | 19.68 | p__*Proteobacteria*; c__*Gammaproteobacteria*; o__*Vibrionales*; f__*Vibrionaceae*; g__*Photobacterium* |
| 03 | 12.46 | p__*Proteobacteria*; c__*Gammaproteobacteria*; o__*Vibrionales*; f__*Vibrionaceae*; g__*Vibrio* |
| 04 | 12.13 | p__*Firmicutes*; c__*Bacilli*; o__*Mycoplasmatales*; f__*Mycoplasmataceae*; g__*Candidatus* Bacilliplasma |
| 05 | 3.64 | p__*Proteobacteria*; c__*Gammaproteobacteria*; o__*Alteromonadales*; f__*Psychromonadaceae*; g__*Motilimonas* |
| 06 | 2.65 | p__*Actinobacteriota*; c__*Actinobacteria*; o__*Corynebacteriales*; f__*Corynebacteriaceae*; g__*Corynebacterium* |
| 07 | 2.54 | p__*Firmicutes*; c__*Bacilli*; o__*Mycoplasmatales*; f__*Mycoplasmataceae*; g__*Candidatus* Bacilliplasma |
| 08 | 2.46 | p__*Proteobacteria*; c__*Alphaproteobacteria*; o__*Rhodobacterales*; f__*Rhodobacteraceae*; g__*Ruegeria* |
| 09 | 1.86 | p__*Proteobacteria*; c__*Alphaproteobacteria*; o__*Rhodobacterales*; f__*Rhodobacteraceae*; g__*Roseovarius* |
| 10 | 1.32 | p__*Firmicutes*; c__*Bacilli*; o__*Staphylococcales*; f__*Staphylococcaceae*; g__*Staphylococcus* |
| 11 | 1.03 | p__*Proteobacteria*; c__*Alphaproteobacteria*; o__*Rhizobiales*; f__*Stappiaceae*; g__*Labrenzia* |
| 12 | 0.93 | p__*Actinobacteriota*; c__*Actinobacteria*; o__*Corynebacteriales*; f__*Corynebacteriaceae*; g__*Corynebacterium* |
| 13 | 0.80 | p__*Proteobacteria*; c__*Alphaproteobacteria*; o__*Rhodobacterales*; f__*Rhodobacteraceae*; g__*Shimia* |
| 14 | 0.80 | p__*Proteobacteria*; c__*Gammaproteobacteria*; o__*Vibrionales*; f__*Vibrionaceae*; g__*Photobacterium* |
| 15 | 0.64 | p__*Actinobacteriota*; c__*Actinobacteria*; o__*Kineosporiales*; f__*Kineosporiaceae* |
| 16 | 0.62 | p__*Actinobacteriota*; c__*Actinobacteria*; o__*Corynebacteriales*; f__*Corynebacteriaceae*; g__*Corynebacterium* |
| 17 | 0.54 | p__*Firmicutes*; c__*Bacilli*; o__*Erysipelotrichales*; f__*Erysipelotrichaceae*; g__ZOR0006 |
| 18 | 0.50 | p__*Proteobacteria*; c__*Alphaproteobacteria*; o__*Rhodobacterales*; f__*Rhodobacteraceae*; g__*Paracoccus* |
| 19 | 0.47 | p__*Firmicutes*; c__*Bacilli*; o__*Mycoplasmatales*; f__*Mycoplasmataceae*; g__*Candidatus* Bacilliplasma |
| 20 | 0.44 | p__*Proteobacteria*; c__*Alphaproteobacteria*; o__*Rhodobacterales*; f__*Rhodobacteraceae* |
| 21 | 0.41 | p__*Proteobacteria*; c__*Alphaproteobacteria*; o__*Rhodobacterales*; f__*Rhodobacteraceae* |
| 22 | 0.35 | p__*Actinobacteriota*; c__*Actinobacteria*; o__*Corynebacteriales*; f__*Corynebacteriaceae*; g__*Corynebacterium* |
| 23 | 0.34 | p__*Verrucomicrobiota*; c__*Verrucomicrobiae*; o__*Verrucomicrobiales*; f__*Rubritaleaceae*; g__*Haloferula* |
| 24 | 0.34 | p__*Firmicutes*; c__*Bacilli*; o__*Mycoplasmatales*; f__*Mycoplasmataceae*; g__*Candidatus* Bacilliplasma |
| 25 | 0.30 | p__*Actinobacteriota*; c__*Actinobacteria*; o__*Corynebacteriales*; f__*Corynebacteriaceae*; g__*Corynebacterium* |
| 26 | 0.30 | p__*Proteobacteria*; c__*Gammaproteobacteria*; o__*Alteromonadales*; f__*Pseudoalteromonadaceae*; g__*Pseudoalteromonas* |
| 27 | 0.27 | p__*Proteobacteria*; c__*Gammaproteobacteria*; o__*Burkholderiales*; f__*Comamonadaceae*; g__*Comamonas* |
| 28 | 0.27 | p__*Bacteroidota*; c__*Bacteroidia*; o__*Flavobacteriales*; f__*Flavobacteriaceae*; g__*Formosa* |
| 29 | 0.26 | p__*Actinobacteriota*; c__*Actinobacteria*; o__*Micrococcales*; f__*Dermacoccaceae*; g__*Kytococcus* |
| 30 | 0.23 | p__*Proteobacteria*; c__*Alphaproteobacteria*; o__*Rhodobacterales*; f__*Rhodobacteraceae* |

**Table S6. Annotation of the top 30 ASVs in shrimp gut microbiota of the S02-infected group.**

| ASV  No. | Average abundance (%) | Taxonomic annotation (phylum to genus) |
| --- | --- | --- |
| 04 | 34.30% | p__*Firmicutes;* c__*Bacilli;* o__*Mycoplasmatales;* f__*Mycoplasmataceae;* g__*Candidatus* Bacilliplasma |
| 01 | 26.48% | p__*Firmicutes;* c__*Bacilli;* o__*Mycoplasmatales;* f__*Mycoplasmataceae;* g__*Candidatus* Bacilliplasma |
| 03 | 5.68% | p__*Proteobacteria;* c__*Gammaproteobacteria;* o__*Vibrionales;* f__*Vibrionaceae;* g__*Vibrio* |
| 08 | 4.34% | p__*Proteobacteria;* c__*Alphaproteobacteria;* o__*Rhodobacterales;* f__*Rhodobacteraceae;* g__*Ruegeria* |
| 02 | 4.28% | p__*Proteobacteria;* c__*Gammaproteobacteria;* o__*Vibrionales;* f__*Vibrionaceae;* g__*Photobacterium* |
| 09 | 3.20% | p__*Proteobacteria;* c__*Alphaproteobacteria;* o__*Rhodobacterales;* f__*Rhodobacteraceae;* g__*Roseovarius* |
| 05 | 2.20% | p__*Proteobacteria;* c__*Gammaproteobacteria;* o__*Alteromonadales;* f__*Psychromonadaceae;* g__*Motilimonas* |
| 06 | 2.13% | p__*Actinobacteriota;* c__*Actinobacteria;* o__*Corynebacteriales;* f__*Corynebacteriaceae;* g__*Corynebacterium* |
| 07 | 1.81% | p__*Firmicutes;* c__*Bacilli;* o__*Mycoplasmatales;* f__*Mycoplasmataceae;* g__*Candidatus* Bacilliplasma |
| 10 | 1.05% | p__*Firmicutes;* c__*Bacilli;* o__*Staphylococcales;* f__*Staphylococcaceae;* g__*Staphylococcus* |
| 11 | 0.87% | p__*Proteobacteria;* c__*Alphaproteobacteria;* o__*Rhizobiales;* f__*Stappiaceae;* g__*Labrenzia* |
| 31 | 0.78% | p__*Proteobacteria;* c__*Alphaproteobacteria;* o__*Rhodobacterales;* f__*Rhodobacteraceae* |
| 22 | 0.66% | p__*Actinobacteriota;* c__*Actinobacteria;* o__*Corynebacteriales;* f__*Corynebacteriaceae;* g__*Corynebacterium* |
| 13 | 0.56% | p__*Proteobacteria;* c__*Alphaproteobacteria;* o__*Rhodobacterales;* f__*Rhodobacteraceae;* g__*Shimia* |
| 17 | 0.52% | p__*Firmicutes;* c__*Bacilli;* o__*Erysipelotrichales;* f__*Erysipelotrichaceae;* g__ZOR0006 |
| 28 | 0.52% | p__*Bacteroidota;* c__*Bacteroidia;* o__*Flavobacteriales;* f__*Flavobacteriaceae;* g__*Formosa* |
| 12 | 0.44% | p__*Actinobacteriota;* c__*Actinobacteria;* o__*Corynebacteriales;* f__*Corynebacteriaceae;* g__*Corynebacterium* |
| 32 | 0.39% | p__*Firmicutes;* c__*Bacilli;* o__*Mycoplasmatales;* f__*Mycoplasmataceae;* g__*Candidatus* Bacilliplasma |
| 19 | 0.31% | p__*Firmicutes;* c__*Bacilli;* o__*Mycoplasmatales;* f__*Mycoplasmataceae;* g__*Candidatus* Bacilliplasma |
| 18 | 0.31% | p__*Proteobacteria;* c__*Alphaproteobacteria;* o__*Rhodobacterales;* f__*Rhodobacteraceae;* g__*Paracoccus* |
| 33 | 0.28% | p__*Proteobacteria;* c__*Alphaproteobacteria;* o__*Rhodobacterales;* f__*Rhodobacteraceae* |
| 20 | 0.25% | p__*Proteobacteria;* c__*Alphaproteobacteria;* o__*Rhodobacterales;* f__*Rhodobacteraceae* |
| 34 | 0.24% | p__*Bacteroidota;* c__*Bacteroidia;* o__*Flavobacteriales;* f__*Flavobacteriaceae;* g__*Tenacibaculum* |
| 35 | 0.24% | p__*Proteobacteria;* c__*Alphaproteobacteria;* o__*Rhodobacterales;* f__*Rhodobacteraceae* |
| 15 | 0.23% | p__*Actinobacteriota;* c__*Actinobacteria;* o__*Kineosporiales;* f__*Kineosporiaceae* |
| 36 | 0.23% | p__*Firmicutes;* c__*Bacilli;* o__*Mycoplasmatales;* f__*Mycoplasmataceae;* g__*Candidatus* Bacilliplasma |
| 24 | 0.21% | p__*Firmicutes;* c__*Bacilli;* o__*Mycoplasmatales;* f__*Mycoplasmataceae;* g__*Candidatus* Bacilliplasma |
| 21 | 0.19% | p__*Proteobacteria;* c__*Alphaproteobacteria;* o__*Rhodobacterales;* f__*Rhodobacteraceae* |
| 37 | 0.19% | p__*Actinobacteriota;* c__*Acidimicrobiia;* o__*Microtrichales;* f__*Microtrichaceae;* g__*uncultured* |
| 38 | 0.18% | p__*Planctomycetota;* c__*Planctomycetes;* o__*Pirellulales;* f__*Pirellulaceae;* g__*Blastopirellula* |

**Table S7. Annotation of the top 30 ASVs in shrimp gut microbiota of the TSB-treated group.**

| ASV  No. | Average abundance (%) | Taxonomic annotation (phylum to genus) |
| --- | --- | --- |
| 04 | 24.86% | p__*Firmicutes;* c__*Bacilli;* o__*Mycoplasmatales;* f__*Mycoplasmataceae;* g__*Candidatus* Bacilliplasma |
| 01 | 20.66% | p__*Firmicutes;* c__*Bacilli;* o__*Mycoplasmatales;* f__*Mycoplasmataceae;* g__*Candidatus* Bacilliplasma |
| 02 | 18.78% | p__*Proteobacteria;* c__*Gammaproteobacteria;* o__*Vibrionales;* f__*Vibrionaceae;* g__*Photobacterium* |
| 03 | 3.93% | p__*Proteobacteria;* c__*Gammaproteobacteria;* o__*Vibrionales;* f__*Vibrionaceae;* g__*Vibrio* |
| 08 | 3.17% | p__*Proteobacteria;* c__*Alphaproteobacteria;* o__*Rhodobacterales;* f__*Rhodobacteraceae;* g__*Ruegeria* |
| 07 | 3.10% | p__*Firmicutes;* c__*Bacilli;* o__*Mycoplasmatales;* f__*Mycoplasmataceae;* g__*Candidatus* Bacilliplasma |
| 06 | 1.86% | p__*Actinobacteriota;* c__*Actinobacteria;* o__*Corynebacteriales;* f__*Corynebacteriaceae;* g__*Corynebacterium* |
| 05 | 1.38% | p__*Proteobacteria;* c__*Gammaproteobacteria;* o__*Alteromonadales;* f__*Psychromonadaceae;* g__*Motilimonas* |
| 19 | 1.28% | p__*Firmicutes;* c__*Bacilli;* o__*Mycoplasmatales;* f__*Mycoplasmataceae;* g__*Candidatus* Bacilliplasma |
| 10 | 1.18% | p__*Firmicutes;* c__*Bacilli;* o__*Staphylococcales;* f__*Staphylococcaceae;* g__*Staphylococcus* |
| 31 | 0.92% | p__*Proteobacteria;* c__*Alphaproteobacteria;* o__*Rhodobacterales;* f__*Rhodobacteraceae* |
| 09 | 0.91% | p__*Proteobacteria;* c__*Alphaproteobacteria;* o__*Rhodobacterales;* f__*Rhodobacteraceae;* g__*Roseovarius* |
| 11 | 0.85% | p__*Proteobacteria;* c__*Alphaproteobacteria;* o__*Rhizobiales;* f__*Stappiaceae;* g__*Labrenzia* |
| 34 | 0.75% | p__*Bacteroidota;* c__*Bacteroidia;* o__*Flavobacteriales;* f__*Flavobacteriaceae;* g__*Tenacibaculum* |
| 18 | 0.73% | p__*Proteobacteria;* c__*Alphaproteobacteria;* o__*Rhodobacterales;* f__*Rhodobacteraceae;* g__*Paracoccus* |
| 13 | 0.68% | p__*Proteobacteria;* c__*Alphaproteobacteria;* o__*Rhodobacterales;* f__*Rhodobacteraceae;* g__*Shimia* |
| 39 | 0.66% | p__*Proteobacteria;* c__*Alphaproteobacteria;* o__*Rhizobiales;* f__*Rhizobiaceae;* g__*Allorhizobium-Neorhizobium-Pararhizobium-Rhizobium* |
| 17 | 0.63% | p__*Firmicutes;* c__*Bacilli;* o__*Erysipelotrichales;* f__*Erysipelotrichaceae;* g__ZOR0006 |
| 14 | 0.50% | p__*Proteobacteria;* c__*Gammaproteobacteria;* o__*Vibrionales;* f__*Vibrionaceae;* g__*Photobacterium* |
| 12 | 0.50% | p__*Actinobacteriota;* c__*Actinobacteria;* o__*Corynebacteriales;* f__*Corynebacteriaceae;* g__*Corynebacterium* |
| 24 | 0.44% | p__*Firmicutes;* c__*Bacilli;* o__*Mycoplasmatales;* f__*Mycoplasmataceae;* g__*Candidatus* Bacilliplasma |
| 22 | 0.32% | p__*Actinobacteriota;* c__*Actinobacteria;* o__*Corynebacteriales;* f__*Corynebacteriaceae;* g__*Corynebacterium* |
| 40 | 0.31% | p__*Proteobacteria;* c__*Alphaproteobacteria;* o__*Rhodobacterales;* f__*Rhodobacteraceae;* g__*Albimonas* |
| 36 | 0.29% | p__*Firmicutes;* c__*Bacilli;* o__*Mycoplasmatales;* f__*Mycoplasmataceae;* g__*Candidatus* Bacilliplasma |
| 41 | 0.28% | p__*Proteobacteria;* c__*Gammaproteobacteria;* o__*Burkholderiales;* f__*Oxalobacteraceae;* g__*Massilia* |
| 42 | 0.26% | p__*Proteobacteria;* c__*Alphaproteobacteria;* o__*Rhodobacterales;* f__*Rhodobacteraceae* |
| 32 | 0.26% | p__*Firmicutes;* c__*Bacilli;* o__*Mycoplasmatales;* f__*Mycoplasmataceae;* g__*Candidatus* Bacilliplasma |
| 21 | 0.26% | p__*Proteobacteria;* c__*Alphaproteobacteria;* o__*Rhodobacterales;* f__*Rhodobacteraceae* |
| 33 | 0.23% | p__*Proteobacteria;* c__*Alphaproteobacteria;* o__*Rhodobacterales;* f__*Rhodobacteraceae* |
| 15 | 0.22% | p__*Actinobacteriota;* c__*Actinobacteria;* o__*Kineosporiales;* f__*Kineosporiaceae* |
